# *GoSAMT*s are required for pectin methyl-esterification and mucilage release in seed coat epidermal cells

**DOI:** 10.3389/fpls.2023.1099573

**Published:** 2023-02-08

**Authors:** Juan Pablo Parra-Rojas, Pablo Sepúlveda-Orellana, Dayan Sanhueza, Hernán Salinas-Grenet, Henry Temple, Paul Dupree, Susana Saez-Aguayo, Ariel Orellana

**Affiliations:** ^1^ Centro de Biotecnología Vegetal, Facultad de Ciencias de la Vida, Universidad Andrés Bello, Santiago, Chile; ^2^ Millenium Institute Center for Genome Regulation, Santiago, Chile; ^3^ Department of Biochemistry, University of Cambridge, Cambridge, United Kingdom

**Keywords:** *GoSAMT (Golgi SAM Transporter)*, seed coat, mucilage, pectin, methyl-esterification, homogalacturonan

## Abstract

**Introduction:**

*GoSAMTs* play a role in the methylation of polysaccharides synthesized by the Golgi. Pectin homogalacturonan (HG) methyl-esterification is essential for the proper function of this polysaccharide in cell walls. In order to better understand the role of *GoSAMTs* in HG biosynthesis, we analyzed mucilage methyl-esterification in *gosamt* mutants.

**Methods:**

To determine the function of *GoSAMT1* and *GoSAMT2* in HG methyl-esterification we utilized epidermal cells of seed coats, as these structures produce mucilage, which is a pectic matrix. We evaluated differences in seed surface morphology and quantified mucilage release. We measured methanol release, and used antibodies and confocal microscopy to analyze HG methyl-esterification in mucilage.

**Results:**

We observed morphological differences on the seed surface and delayed, uneven mucilage release in *gosamt1-1gosamt2-1* double mutants. We also found changes in the distal wall length indicating abnormal cell wall breakage in this double mutant. Using methanol release and immunolabeling, we confirmed that *GoSAMT1* and *GoSAMT2* are involved in HG methyl-esterification in mucilage. However, we did not find evidence of decreasing HG in the *gosamt* mutants. Confocal microscopy analyses detected different patterns in the adherent mucilage and a greater number of low-methyl-esterified domains near the seed coat surface, which correlates with a greater number of “egg-box” structures in this region. We also detected a shift in the partitioning between the Rhamnogalacturonan-I soluble and adherent layers of the double mutant, which correlated with increased amounts of arabinose and arabinogalactan-protein in the adherent mucilage.

**Discussion:**

The results show that the HG synthesized in *gosamt* mutant plants is less methyl esterified, resulting in more egg-box structures, which stiffen the cell walls in epidermal cells and change the rheological properties of the seed surface. The increased amounts of arabinose and arabinogalactan-protein in adherent mucilage, also suggests that compensation mechanisms were triggered in the *gosamt* mutants.

## 1 Introduction

Homogalacturonan (HG) is the most abundant pectin domain in the primary plant cell wall and plays several roles in plant physiology and development. It is composed of a linear chain of galacturonic acid (GalA) with different degrees of methyl-esterification, a critical feature that controls cell adhesion and expansion, as well as interactions with other macromolecules (Sénéchal et al., 2014; Francoz et al., 2019; Du et al., 2022a); methyl-esterification occurs while HG is biosynthesized in the Golgi apparatus by methyltransferases (Zhang and Staehelin, 1992). Kim et al. (2015) documented *CGR2* and *CGR3* (i.e., *Cotton-Golgi-Related 2* and *Cotton-Golgi-Related 3*) gene-coding for putative methyl-transferases involved in HG methyl-esterification; and more recently, Du et al. (2020) observed *QUASIMODO 2* (*QUA2*) coding for an HG methyl-transferase. Notably, *QUA2* mutants demonstrated defective cell-to-cell adhesion and reduced HG content, which underscores the importance of pectin methyl-esterases in HG regulation (Mouille et al., 2007; Du et al., 2020).

The HG–methyl-transferase catalytic domain faces the Golgi lumen and utilizes S-Adenosylmethionine (SAM) as a substrate (Ibar and Orellana, 2007); SAM is synthesized in the cytosol and transported into the Golgi apparatus by Golgi SAM Transporters (*GoSAMT*s) (Ibar and Orellana, 2007; Temple et al., 2022). A three-member family of Major Facilitator Superfamily 5 (MFS 5) coding for *GoSAMT* was identified in 2022, and mutants in these genes exhibited reduced methylation in HG and Xylan (i.e., GlcA in xylan), which confirms SAM must be transported into the Golgi lumen for HG methyl-esterification (Temple et al., 2022). After biosynthesis, HG is secreted onto the cell wall, where it becomes demethyl-esterified by pectin methyl esterase activities (PMEs), which are regulated by pectin methyl esterase inhibitors (PMEIs) (Peaucelle et al., 2011). In this way, HG demethyl-esterification is subjected to finely tuned PME- and PMEI-based regulations. Wang et al. (2013) determined that *Arabidopsis* has 66 PME-coding genes and 71 PMEI-coding genes, which supports Levesque-Tremblay et al. (2015), who asserted HG demethyl-esterification is a highly regulated process.

*Arabidopsis* mucilage is a pectin-rich matrix, making it an viable model to study HG methyl-esterification (Saez-Aguayo et al., 2013; Turbant et al., 2016; Šola et al., 2019; Ding et al., 2021). Mucilage is produced in the epidermal cells of seed coats, the cell walls of which break open after imbibition and release mucilage (Western et al., 2000; Macquet et al., 2007); notably, mutants in HG methyl-esterification exhibit a delayed release of mucilage (Saez-Aguayo et al., 2013), which suggests the epidermal cell breakage was altered. Seed mucilage is comprised of two layers: The soluble mucilage (SM) layer becomes detached by shaking the seeds in water, and the adherent mucilage (AM) layer becomes evident after Ruthenium red staining; both layers are primarily composed of rhamnogalacturonan-I (RG-I) (Macquet et al., 2007; Arsovski et al., 2010). It is worth noting that several studies have asserted HG only accounts for approximately 10% of the polysaccharides in mucilage (Voiniciuc et al., 2015; Šola et al., 2019). Even though the levels of certain polysaccharides (i.e., xylan, galactomannan, and xyloglucan) have been found to be lower than HG in some studies, evidence of methyl-esterification in any of the polysaccharides in mucilage other than HG has not yet been produced (Voiniciuc et al., 2015; Šola et al., 2019).

In addition to PME and PMEI gene-coding mutants (i.e., *PME58*, *HMS/PME6*; and *PMEI6*); transcription-factor mutants *STK*, *MYB52*, and *MUM1* also regulate PME and PMEI expression and have been pivotal to properly understanding the role of methyl-esterification in seed coat mucilage (Saez-Aguayo et al., 2013; Levesque-Tremblay et al., 2015; Ezquer et al., 2016; Turbant et al., 2016; Shi et al., 2018; Ding et al., 2021). Furthermore, *SBT1.7* could be involved in PME and PMEI modification and regulation and therefore contributes to cell wall properties (Rautengarten et al., 2008); finally, mutants in *FLY1*, a gene involved in recycling cell-wall-modifying enzymes, also exhibit lower methyl-esterification (Voiniciuc et al., 2013). These studies provide additional information that confirms the importance of HG methyl-esterification on mucilage extrusion and mucilage partitioning. Furthermore, a decrease in methyl-esterification may also reduce overall HG content. Interestingly, recent studies demonstrated PMEI6-produced demethyl-esterification patterns in mucilage, which is needed to position and anchor PRX36, a peroxidase involved in mucilage release that triggers cell wall loosening during development (Saez-Aguayo et al., 2013; Francoz et al., 2019). These results not only demonstrate the importance of the degree of post-synthesis HG methyl-esterification, but also reveal that a pattern of methyl-esterification domains are formed by PMEs and PMEIs.

Another important player in HG methyl-esterification is pectin methyl-transferases (PMTs). Du et al. (2022b) reported that *QUA2*, a pectin methyl-transferase involved in HG methyl-esterification in the Golgi apparatus, is involved in mucilage formation. Furthermore, the *tumorous shoot development2* (*tsd2*) and *things fall apart2* (*tfa2*) *QUA2* mutant alleles yielded lower uronic acid content and released less methanol from mucilage (Du et al., 2022b).

The identification of *GoSAMT*s provides an unprecedented new tool to study methylation, because we can now investigate the impact thereof on a process occurring upstream in HG methyl-esterification compared to the role of PMEs, PMEIs, and PMTs. As such, we decided to study the effect of altered *GoSAMT*s on mucilage formation using mutants of these genes. We hypothesized that mutants in these transporters would incorporate fewer methyl groups on the HG in the Golgi; and we anticipated HG secreted in the mutants should be under-methyl-esterified and might contain different methyl-esterification patterns than those of the wild-type. We observed *GoSAMT1* is the most expressed gene in seeds, followed by *GoSAMT2*, then *GoSAMT3*. The *gosamt1* mutants exhibited a delayed extrusion of mucilage, and this was even more noticeable in the *gosamt1-1gosamt2-1* double mutant.

In addition to revealing morphological changes in the radial epidermal cell walls of this double mutant and differences in epidermal cell-wall breakage after imbibition, we also detected a notable decrease in methanol released from mucilage consistent with decreased HG methyl-esterification when antibodies are used; interestingly, we did not observe a significant change in the HG levels of the wild-type and *gosamt1-1gosamt2-1*. Furthermore, immunolabeling experiments followed by confocal analyses revealed significant changes in the distribution of the antibody-detected epitopes, which suggests the location of HG methyl-esterification domains in the AM layer changed. While the monosaccharide composition analyses revealed decreased levels of GalA, Rhamnose (Rha), and Xylose (Xyl) in the SM layer; the AM layer conversely exhibited increased levels of GalA, Rha, and Xyl. This suggests the RG-I partitioning between each layer was altered, which correlates with the increased levels of arabinose (Ara) and arabinogalactan proteins (AGP) detected by the LM30 antibody in the AM layer. Our findings demonstrate that decreasing the SAM supply during HG biosynthesis alters HG methyl-esterification and leads to the formation of a greater number of “egg-box”-like structures in the seed coat mucilage, resulting in stiffer distal epidermal cell-wall that alter epidermal-cell breakage and delay mucilage extrusion.

## 2 Materials and methods

### 2.1 Plant growth

*Arabidopsis thaliana* ecotype Columbia-0 (Col-0) was utilized as a wild-type plant for all T-DNA insertion lines. These plants were grown in soil (Top Crop^™^); supplemented with fertilizer (Top Crop^™^); and maintained in a growth chamber under a 16-hour photoperiod (i.e., a long day) with 120 μmol m^−2^ s^−1^ light intensity, a temperature ranging between 19–24°C, and 65% relative humidity. The following T-DNA insertion lines were analyzed in this study: *gosamt1-1* (SALK_054431); *gosamt1-2* (SALKseq_038644); *gosamt2-1* (GK_480G06); *gosamt2-2* (SALK_045404); *gosamt3-1* (SALK_105511); and *gosamt3-2* (SALK_077357). The *gosamt1-1*, *gosamt2-1*, and *gosamt3-1* T-DNA lines were provided by Dr. Paul Dupree (Department of Biochemistry, University of Cambridge, Cambridge, UK) and published by Temple et al. (2022); the *gosamt1-1gosamt2-1*, *gosamt1-1gosamt3-1*, and *gosamt2-1gosamt3-1* double-mutant plants were generated and analyzed in this study; and the remaining mutant lines were obtained from ABRC (http://abrc.osu.edu) using SIGnAL Salk collection and selected by PCR (Alonso et al., 2003).

#### 2.1.1 Mucilage extrusion analyses using Ruthenium red staining

Mucilage released from the mature dry seeds was stained with 0.01% w/v Ruthenium red; then the mucilage extrusion was observed and live-captured with a LEICA EZ4 HD stereoscopic microscope (Leica, Wetzlar, Germany). This experiment was performed using 20 seeds of each genotype and four biological replicates.

#### 2.1.2 Determining epidermal cell distal-wall phenotypes

ImageJ 1.53 software (Freeware, National Institute of Health) was employed to measure the length of the epidermal cell distal wall (dw); in this endeavor, imbibed mature seeds were stained with Pontamine fast scarlet S4B, then measured from the top of the columella to the end of the dw. After measuring the mean WT Col-0 dw, we defined three groups of phenotypes using a +/−15% function range of the calculated WT Col-0 mean: Short (S) dw phenotypes were more than 15% below the mean, long (L) dw phenotypes were more than 15% above the mean, and mean (m) dw phenotypes ranged between −15% and +15% of the mean. Finally, we analyzed 300–450 dw phenotypes from the four biological replicates.

#### 2.1.3 Immunolabeling adherent mucilage

Immunolabeling was performed using six monoclonal antibodies that recognize RG-I and HG epitopes. The INRA RU1 antibody was used to label RG-I; and the JIM5, LM19, JIM7, LM20, and 2F4 antibodies were employed to detect HG in different structures and degrees of methyl-esterification, because each recognizes poorly methyl-esterified, partially and non-methyl-esterified, highly methyl-esterified HG, and egg-box structures (Willats et al., 2000; Verhertbruggen et al., 2009; Ralet et al., 2010; Saez-Aguayo et al., 2013; Saez-Aguayo et al., 2017). We stained the seed surfaces with Propidium iodide (20 mg mL^−1^). To observe cellulose-array organization, we stained the seeds with Calcofluor White (0.01% w/v) and Pontamine fast scarlet S4B (0.01% w/v in 50 mM of NaCl). Images from optical sections were obtained using a Leica TCS LSI spectral confocal laser scanning microscope. A 488 nm argon laser line was used to excite Alexa Fluor 488 and Pontamine fast scarlet S4B; a 405 nm diode laser line was used to excite Calcofluor White; and a 543 nm neon laser line was used to excite the propidium iodide. Fluorescence emission was detected between 504–579 nm for Alexa Fluor 488; between 560–605 nm for Pontamine fast scarlet S4B; between 412–490 nm for Calcofluor White; and between 550–725 nm for propidium iodide. The laser gain value was fixed to allow signal intensities to be compared.

#### 2.1.4 Extraction of soluble and adherent mucilage layers

We performed a sequential extraction to determine the monosaccharide content of the SM and AM layers. To extract the mucilage layers, 30 mg of dry seeds for each genotype was imbibed twice in 4 mL of water for 30 minutes with gentle agitation. Supernatants were recovered after centrifugation and pooled to obtain the SM fraction. To obtain the AM layer, the seeds were imbibed in 2 mL of water, then subjected to sonication from an ultrasonic homogenizer sonic ruptor 250 (Omni International^©^) following the instructions delineated by Parra-Rojas et al. (2019). The detached mucilage was collected by recovering supernatant after centrifugation, rinsing the seeds with 2 mL of water, and pooling the supernatants to obtain the AM fraction. After the soluble and adherent mucilage was extracted, the samples were stabilized by heating them at 100°C for five minutes, then freeze-drying them; finally, the samples were resuspended in deionized water and stored at −20°C. A volume of 50 µL of the soluble and adherent mucilage fractions were hydrolyzed for 35 minutes with 400 µL of 2 M TFA at 121°C using allose and myo-Inositol (250 µM each) as internal standards; the TFA was evaporated at 45°C with gaseous nitrogen, and the samples were rinsed twice in 400 µL of isopropanol and dried with gaseous nitrogen; then the dried samples were suspended in water, filtered through a 0.22 µm pore size, and collected for the HPAEC-PAD analysis.

### 2.2 HPAEC-PAD analysis

Monosaccharide quantification was carried out in a Dionex ICS3000 ion chromatography system (Thermo-Fisher; Waltham, Massachusetts, USA) equipped with a pulsed amperometric detector, a CarboPac PA1 (4 × 250 mm) analytical column, and a CarboPac PA1 (4 × 50 mm) guard column (Thermo-Fisher; Waltham, Massachusetts, USA). Neutral sugar separation was performed at 35°C with a 1 mL min^–1^ flow rate using an isocratic gradient of 18 mM NaOH for 27 minutes, followed by a separation of acidic sugars using 105 mM NaOAc and 120 mM NaOH for eight minutes at a 1 mL min^–1^ flow rate at 35°C and a wash with 200 mM NaOH for five minutes. The column was equilibrated in 18 mM NaOH for 10 minutes after every step; and standard curves of either neutral sugars (i.e., d-Fuc, l-Rha, l-Ara, d-Gal, d-Glc, d-Xyl, and d-Man) or acidic sugars (i.e., d-GalA and d-GlcA) were used for quantification.

#### 2.2.1 Quantitative real-time PCR (RT-qPCR) and RT-PCR expression analysis

Three siliques were dissected 6, 8, 10, and 12 days after pollination (DAP) for further RNA extraction. Total RNA extractions were performed with an RNeasy^®^ Plus Micro Kit (Qiagen) following the manufacturer’s instructions. A 1 μg aliquot of total RNA was treated with 1 µL of Dnase I (Thermo Fisher Scientific) and used as a template for first-strand cDNA synthesis with an oligo(dT) primer and SuperScript II (Thermo Fisher Scientific) according to the manufacturer’s instructions. Fast EvaGreenR qPCR Master Mix (Biotium) was used for the RT-qPCR analysis, and the reactions were performed in a total 10 µL volume. The primers listed in **Supplementary Table 1** were used to amplify each *GoSAMT*. *EF1aA4* and *At4g12590* were used as reference genes (North et al., 2007; Hong et al., 2010). Relative quantification was performed according to Parra-Rojas et al. (2019); the synthesized cDNA was also used for the RT-PCR experiments; and all reactions were carried out with the Sapphire enzyme (Takara, Japan) according to the manufacturer’s instructions. The primers used to amplify the full-length for each gene are described in **Supplementary Table 1**.

#### 2.2.2 Determining methanol content of both mucilage layers

50 µL of SM and AM were saponified with 50 µL of NaOH (0.2 M) at 4°C on ice for one hour to demethyl-esterify pectin polysaccharides. The reaction was stopped by adding 50 µL of HCl (0.2 M) and then 150 µL of ultrapure water was added to dilute the salts formed. A volume of 50 µL of saponified mucilage solution was incubated with 100 µL of Tris-HCl (200 mM at pH 7.5), 40 µL of 3-Methyl-2-benzothiazolinone hydrazone/MBTH (3 mg mL^−1^), and 20 µL of Alcohol oxidase from Pichia pastoris at 0.02U µL^−1^ (Sigma A-2404); then incubated again for 20 minutes at 30°C. To reveal methanol content, it was added to previous mix 200 µL of Sulfamic Acid (Sigma) and Ammonium ferric sulfate dodecahydrate (Sigma) (1:1 w/w in water) and incubated at room temperature for 20 minutes. Then 600 µL of ultrapure water were added and DO were measured at 620. Methanol contents were determined using a standard 0–10 ug µL^−1^ curve of methanol. A minimum of five technical replicates were conducted for each biological repeat as published by Saez-Aguayo et al. (2013).

#### 2.2.3 Global PME activity

To evaluate the global PME activity, ~30 mg of dry seeds was ground up and added to liquid nitrogen, then incubated for one hour at 4°C with an extraction buffer (1 M NaCl, 12.5 mM citric acid, and 50 mM Na_2_HPO_4_ with pH 6.5). The resulting homogenate was centrifuged at 8,000 rpm for five minutes at 4°C in an eppendorf^®^ (Germany) centrifuge model 5804 R with an F45-30-11 rotor; after which the supernatant was collected and centrifuged again to obtain a clean supernatant. Total protein quantification was carried out using a BCA kit (Pierce™ BCA Protein Assay Kit, Thermo Scientific). Equal protein samples (30 µg in 20 µL) were loaded in 4 mm diameter wells in a PME activity matrix containing 50 mL of the gel prepared with 0.1% w/v of apple pectin (Poly-D-galacturonic acid methyl ester with 50–75% esterified pectin, Sigma-Aldrich, product code 93854-100G), 1% w/v agarose, 12.5 mM of citric acid, and 50 mM of Na_2_HPO_4_ with pH 6.5; then incubated overnight at 28°C. After incubation, the plates were stained with 30 mL of Ruthenium red solution at 0.01% w/v for 50 minutes, then washed three times with water for 30 minutes. Finally, the stained areas were analyzed with ImageJ 1.53 software (Freeware, National Institute of Health).

#### 2.2.4 Scanning electron microscopy

Whole mount of mature dry seeds from WT Col-0 and the *gosamt1-1gosamt1-2* mutant line were attached to double-sided carbon sealing tape and coated with gold in a 108 manual sputter coater (Ted Pella Inc.). Morphological details of the seeds surface were photographed using a scanning electron microscope (Hitachi TM3000 Tabletop SEM) set to 15 kV. A minimum of three biological replicates were used for each genotype.

#### 2.2.5 Mucilage immunodot blot assay

Immuno-dot blot assays were performed using SM and AM fractions. JIM7, 2F4, and LM30 monoclonal antibodies were used; the latter was employed because it specifically recognizes arabinogalactan-protein (AGP). A volume of 0.7 µL of either SM or AM fractions were blotted onto a 0.45 µm nitrocellulose membrane (Thermos Scientific, cat. no. 88018). The 2F4-analyzed samples were de-methyl-esterified by an alkali treatment (NaOH 0.2 M) prior to being loaded in the nitrocellulose membrane. The blotted membranes were then incubated with a blocking solution containing 3% w/v of powdered skim milk dissolved in TBS supplemented with 0.1% w/v of Tween-20 (TBS-T). JIM7 and LM30 were diluted to 1:50 in TBS-T with 1% w/v skim milk, and the membranes were incubated for two hours at room temperature (RT); and the 2F4 antibody was diluted to 1:50 in TCaNa buffer per the manufacturers’ recommendation, then supplemented with 1% w/v of skim milk and 0.1% w/v of TBS-T. After being incubated with the primary antibody, the membranes were washed three times with TBS and incubated for 1.5 hours at RT with the corresponding secondary antibody diluted to 1:2,000 in TBS-T. Finally, the membranes were washed and incubated with BCIP/NBT 1 Step chromogenic AP substrate developing solution (Thermo Scientific, cat. no. 34042). The reaction was stopped by being washed three times with distilled water, and the dot signals were semi-quantified using ImageJ 1.53 software (Freeware, National Institute of Health). A minimum of three technical replicates were used for the immunoblot of each SM and AM fraction.

## 3 Results

### 3.1 *GoSAMT*s are expressed in epidermal cells seed coat when mucilage is synthesized

**Figure 1:**
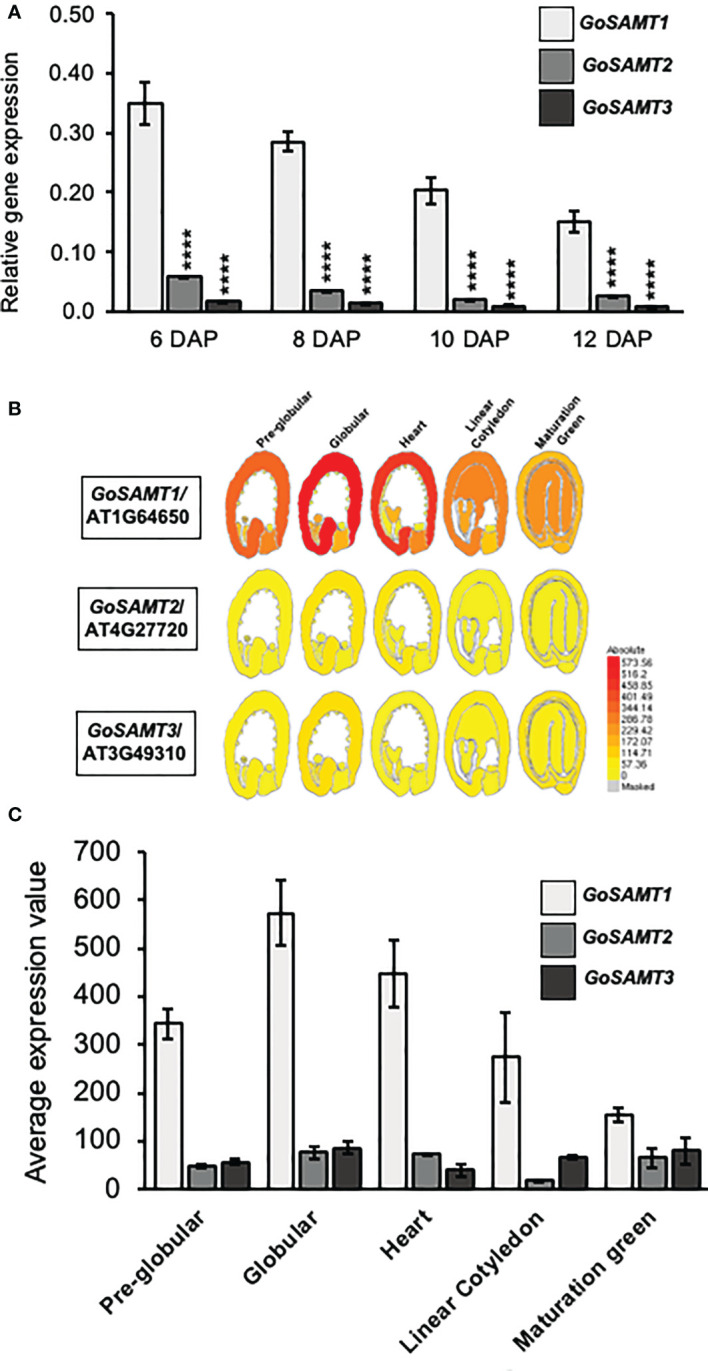
*GoSAMTS* expression pattern in *Arabidopsis thaliana* developing seed. **(A)** qRT-PCR analysis for steady-state levels of *GoSAMTS* gene transcript in developing seeds from 6 to 12 DAP in WT Col-0. Expression was calculated relative to *EFIa44* and an At4g12590 *seed-specific reference gene*. Error bars represent SE values from three biological replicates (*n* = 9). Asterisks indicate significant statistical differences using Unpaired t-test with ****p<0.0001 comparing *GoSAMT1* against *GoSAMT2* and *GoSAMT3* in each stage. **(B)** *GoSAMT* family member expression patterns during seed development; data obtained from eFP browser (http://bar.utoronto.ca/efp_arabidopsis/cgi-bin/efpWeb.cgi) (Winter et al., 2007). **(C)** Average seed coat expression values of *GoSAMT* family members in different seed-developmental stages; data obtained from eFP browser.

Previous studies demonstrated that the *GoSAMTs* code for transporters involved in polysaccharide methyl-esterification; they likely deliver S-Adenosylmethionine, the precursor for pectin methylation, into the Golgi apparatus (Temple et al., 2022). To investigate the participation of these transporters in mucilage HG methylesterification, we initially analyzed the expression thereof throughout seed coat development (**Figure 1A**). *GoSAMT1* exhibited the highest expression in seeds; followed by *GoSAMT2*, the expression of which was approximately five times lower than that of *GoSAMT1*; and finally, *GoSAMT3* was expressed to a much lesser extent with an expression that was approximately 10 times lower than that of *GoSAMT1* (see **Figures 1B, C**). These findings align with the *GoSAMT* seed coat expression data available on the eFPBrowser and supports that *GoSAMT*s are involved in mucilage biosynthesis (Winter et al., 2007; Bassel et al., 2008). Moreover, these results infer *GoSAMT1* may play a leading role in HG methylation, and *GoSAMT2* and *GoSAMT3* may also contribute to varying degrees.

### 3.2 *GoSAMT* mutants exhibit slow-release mucilage phenotype

**Figure 2:**
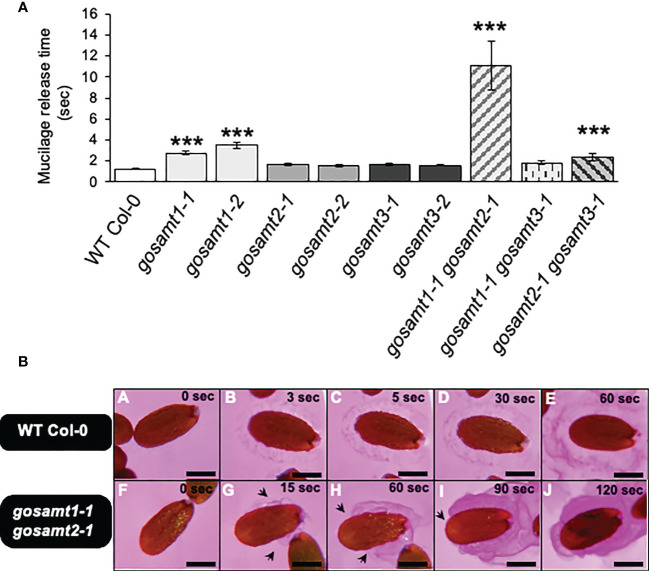
Mucilage release onset time of wild-type and *gosamts* mutants dry seeds. **(A)** Timing of mucilage release performed on mature seeds incubated in a Ruthenium red solution at 21°C. Error bars represent SE for four biological replicates (*n* = 80). Statistical analysis of single comparisons against WT Col-0 performed by Mann-Whitney test (***P < 0.001). **(B)** Images of mucilage release progression in Ruthenium red recorded in WT Col-0 (i.e., A–E) and *gosamt1-1gosamt2-1* double mutant (i.e.,F–J); arrows indicate partial extrusion of soluble mucilage. Scale bars: 200 μm.

Previous reports associated diminished HG methyl-esterification with altered mucilage extrusion (Saez-Aguayo et al., 2013; Du et al., 2020). To determine whether mucilage extrusion was altered in *gosamt* mutant seeds, we therefore evaluated the release of soluble mucilage when seeds are exposed to Ruthenium red (see **Figure 2**). We initially determined the onset of mucilage extrusion in wild-type seeds and in each *gosamt* single mutant. We analyzed two alleles for all single mutants and for the *gosamt1-1gosamt2-1*, *gosamt1-1gosamt3-1*, and *gosamt2-1gosamt3-1* double mutants (see **Figures S1A, B**). Single and double mutants were selected *via* PCR, and RT-PCR was conducted to confirm the T-DNA insertion in each gene (see **Figure S1B**); we did not evaluate triple mutants in the present study because they are lethal (Temple et al., 2022).

Significant differences were observed with two *gosamt1* alleles that demonstrated a 2–3-fold delay in extrusion (see **Figures 2A, B**); this delay increased approximately 10-fold in the *gosamt1-1gosamt2-1* double mutant, compared to the wild-type. Moreover, 65% of the *gosamt1-1gosamt2-1* seeds extruded mucilage, compared to 96% of the wild-type (see **Supplementary Table 2**). Since we detected a notable difference between the onset of the mucilage extrusion of the wild-type and *gosamt1-1gosamt2-1*, we further analyzed the mucilage releases. Whereas the wild-type seeds showed uniform mucilage release; *gosamt1-1gosamt2-1* exhibited a non-uniform extrusion pattern throughout the seed, and uneven mucilage extrusion was also detected (see **Figure 2B**); despite these differences, however, the extrusion in the SM layer was completed in the double mutant after a few minutes of staining incubation. The AM layer, which was observed after full extrusion using Ruthenium red, showed no apparent differences between the wild-type and *gosamt1-1gosamt2-1*, even though a slightly higher labeling was observed in the double mutant. Furthermore, the INRA-RU1 antibody, which labeled a minimum of six Rha-GalA disaccharides in RG-I, revealed no significant differences between the wild-type and the mutants (see **Figure S2**) (Ralet et al., 2010).

### 3.3 Morphological differences of epidermal cells observed in*gosamt1-1gosamt2-1*

**Figure 3:**
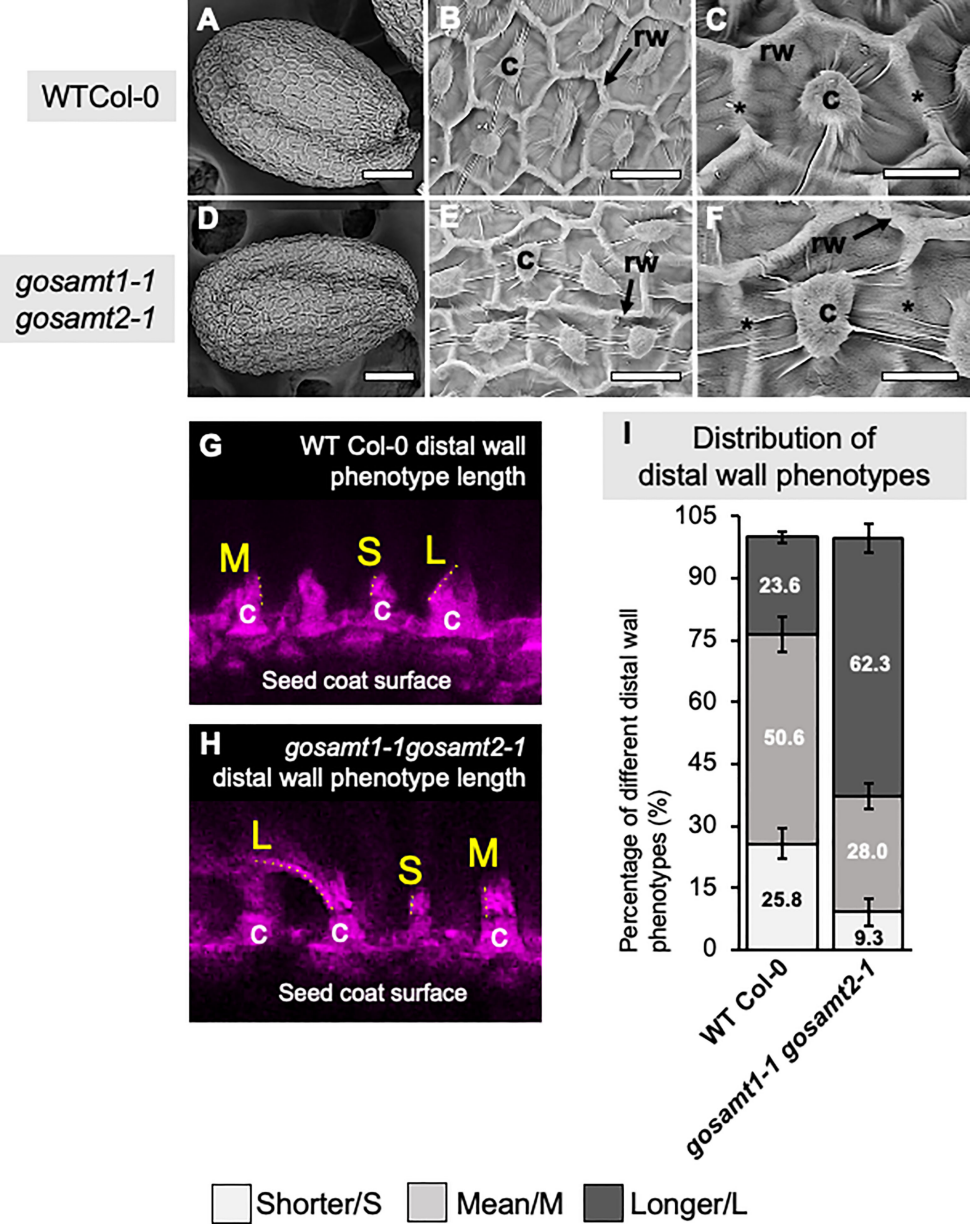
*gosamt1-1gosamt2-1* seed coat epidermal cell morphology is affected and cell-wall breakage is altered. **(A–F)** Scanning electron micrographs (SEM) of WT-Col-0 and *gosamt1-1gosamt2-1* seeds surfaces under different magnifications. Scale bars A and D = 100 μm, B and E 25 µm, and C and F= 10 µM; stars indicate differences between WT Col-0 and the *gosamt1-1gosamt2-1* mutant line. **(G, H)** Images of different distal wall phenotypes after breakage (i.e., S = short; M = mean; L = long) stained with Pontamine fast scarlet (pink). **(I)** Percentage quantification of S, M, and L distal-wall phenotype in *gosamt* double mutant compared to WT Col-0. Error bars represent SE from three biological replicates (*n* = 300–450).

Since the *gosamt1-1gosamt2-1* genotype exhibited the most severe mucilage-extrusion phenotype, we performed scanning electron microscopy of seeds to determine whether there were differences in the seed surface morphology of the double mutant, and we observed the epidermal cells of the *gosamt1-1gosamt2-1* seed coats were more misshapen than those of the wild-type, and gaps in the radial cell wall were more evident in the double mutant than in the wild-type (see **Figures 3A–F**). We also analyzed the distal cell walls that were formed after water imbibition by measuring their length from the top of the columella, and we were able to differentiate long (L), mean (M), and short (S) distal cell walls (see **Figures 3G, H**). While the higher-frequency distal walls in the wild-type corresponded to M, the *gosamt1-1gosamt2-1* double mutant produced high-frequency L distal walls; this infers the primary walls are stronger in the double mutant and break closer to the columella, instead of along the middle as the wild-type does (see **Figure 3I**).

### 3.4 Altered mucilage methyl-esterification in *gosamt* mutants

**Figure 4:**
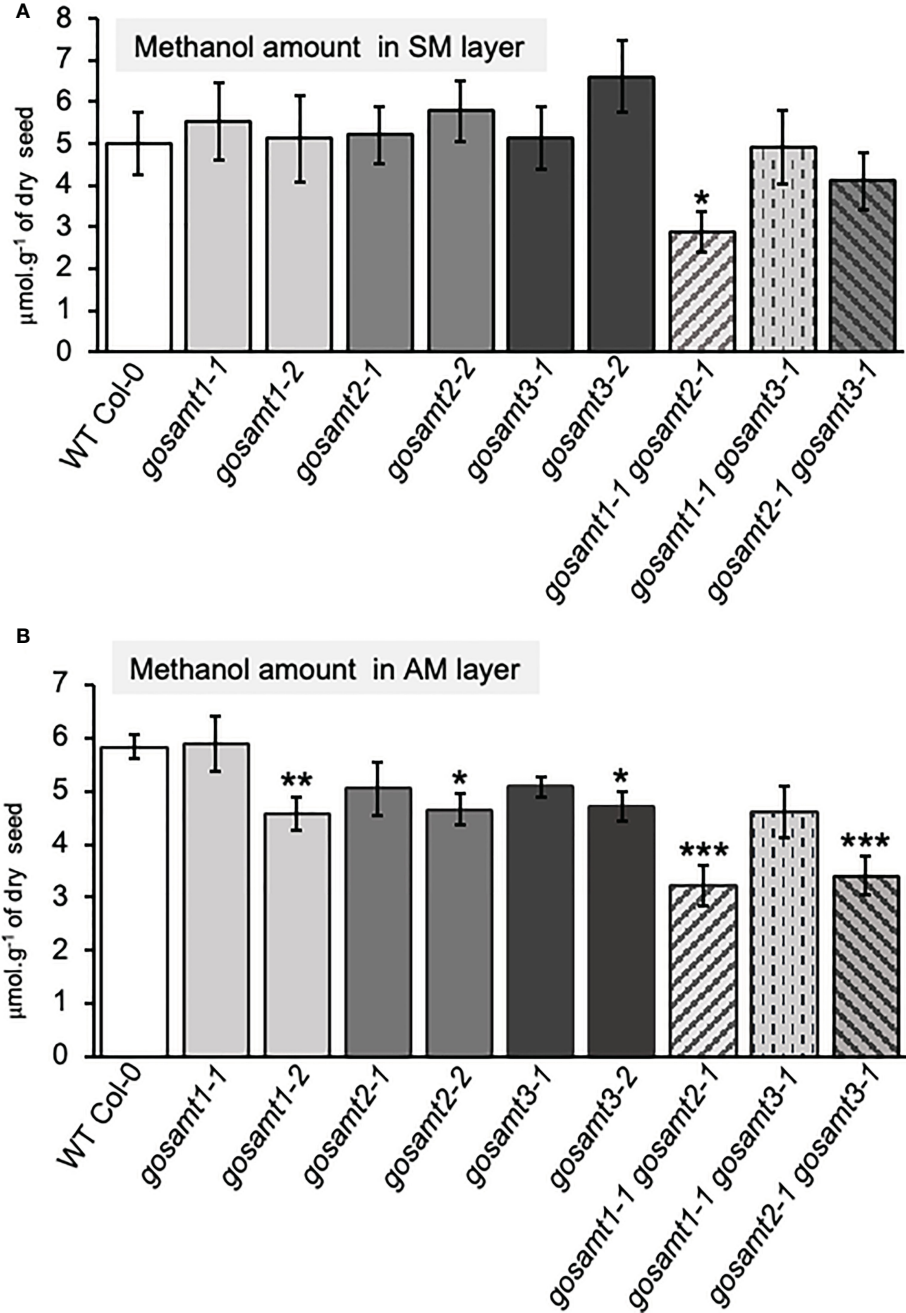
Methanol content of *gosamts* single and double mutants mucilage layers is altered in comparison to the wild-type. Methanol content in soluble **(A)** and adherent **(B)** mucilage layers determined after saponification. Error bars represent SE from five technical replicates of three biological replicates (*n* = 15); asterisks indicate significant statistical differences detected by *t*-test to compare mutant lines with their respective wild-type (*P < 0.05; **P < 0.01, ***P < 0.001).

To evaluate the impact of lacking *GoSAMT*s in mucilage methyl-esterification, we evaluated the methanol content in the soluble and adherent mucilage by measuring the release of methanol (see **Figure 4**). We did not observe any significant differences between the wild-type and all *gosamt* single mutants in the soluble layer (see **Figure 4A**). However, the *gosamt1-1gosamt2-1* double mutant released a lower amount of methanol in the soluble mucilage. The AM layer analysis revealed a slight decrease in the methanol release of most single-mutant alleles, and the *gosamt2-1gosamt3-1* also exhibited reduced methanol release; notably, significantly decreased methanol release was observed in the *gosamt1-1gosamt2-1* seeds (see **Figure 4B**). To determine whether lower HG methyl-esterification resulted in less methanol being released, we performed dot-blot analyses of the SM and AM layers using the JIM7 antibody; and we concluded the *gosamt1-1gosamt2-1* SM and AM layers both produced a weaker signal (see **Figures S3A, B**), which confirms the double mutants contained less methyl-esterified HG.

To determine whether the lower HG methyl-esterification was due to lower HG content in the mutant, we conducted two approaches to estimate the HG content in mucilage. Because RG-I is the primary component of mucilage with a GalA-to-Rha molar ratio equal to 1, we initially estimated the excess GalA moles in relationship to the Rha moles and made the assumption that this remnant pool of GalA would account for the HG content; but we did not observe any significant differences between the wild-type and the *gosamt1-1gosamt2-1* double mutant (see **Figure S4**). We then developed a second approach in which all methyl groups were eliminated from the HG, followed by a dot-blot analysis of the unmethyl-esterified HG using the 2F4 antibody, which can detect non-methyl-esterified HG (Liners et al., 1992); this assessment also failed to reveal differences between the wild-type and the double mutant (see **Figures S3A, B**). After considering these findings together, we concluded that while *gosamt1-1gosamt2-1* seeds produce less methyl-esterified HG, there was no significant change in their HG content.

**Figure 5:**
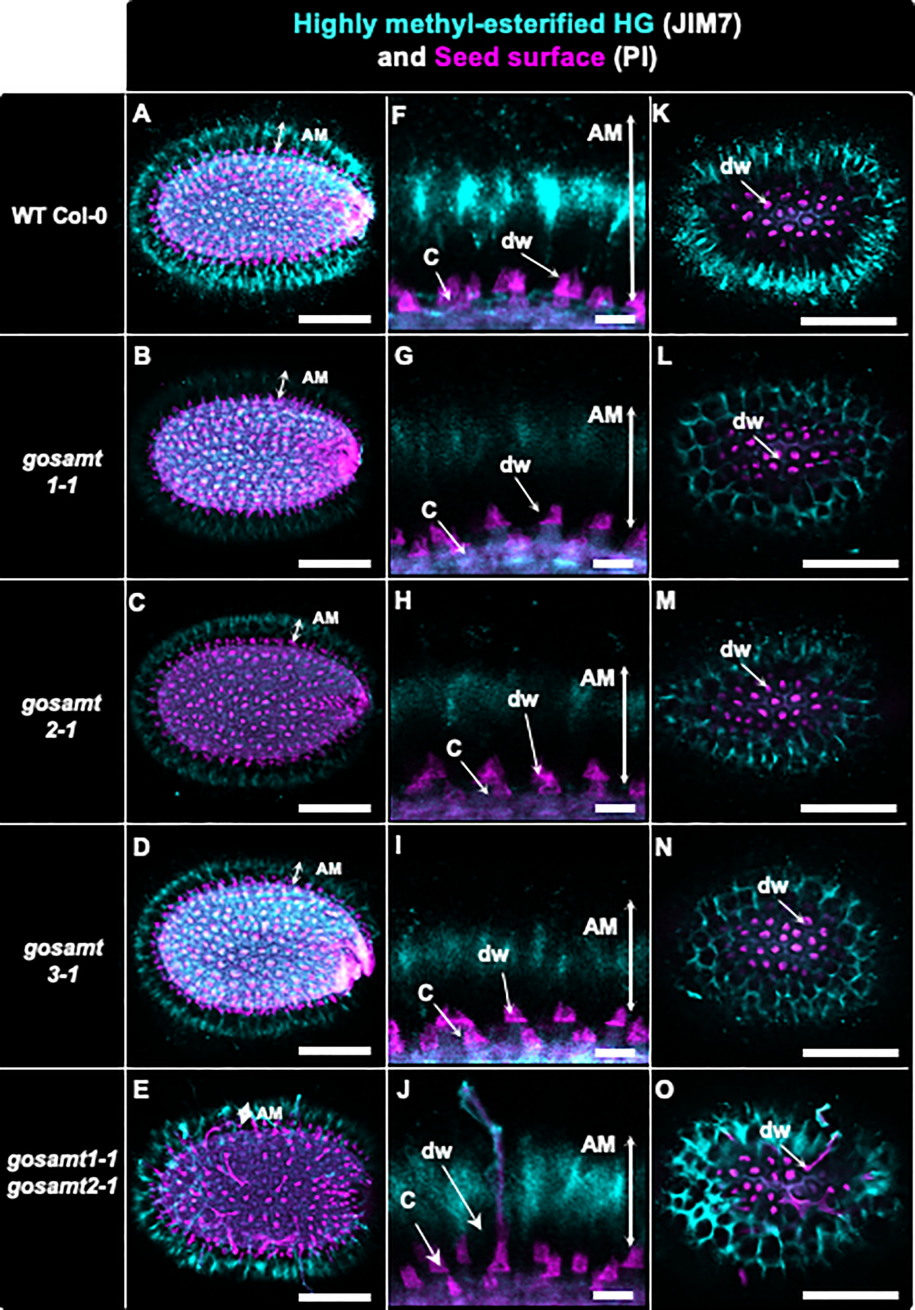
Distribution of highly methyl-esterified homogalacturonans recognized by JIM7 antibody in adherent mucilage layers of wild-type and *gosamts* mutants. Confocal microscopy images of AM released from WT Col-0 and *gosamts* mutants mature imbibed seeds. Fluorescence corresponds to JIM7 antibody labelling in cyan, and propidium iodide, used to detect the seed surface in magenta. **(A–E)** Different optical planes showing entire seed. **(F–J)** Close-up of columella. **(K–O)** Top view of seed surface. C = columella; dw = distal cell wall; vertical arrows in **(F–J)** indicate labelled AM. Scale bars: **(A–E)** and **(K–O)** = 200 µm; **(F–J)**=50 µm.

To further evaluate the altered HG methylation in the AM layer, we performed immunofluorescence of the seeds using JIM7, followed by confocal microscopy analyses (see **Figure 5**); the signal detected in the *gosamt1* and *gosamt2* seed mucilage was lower than that of the wild-type, which indicates HG methylation in the AM layer of these mutants (see **Figure 5**); in contrast, only a slight decrease was observed in seeds from the *gosamt3* mutant lines. While labelling in seeds from the *gosamt1-1gosamt2-1* double mutant was lower than the wild-type, the fluorescence pattern was different, and the epitopes recognized by JIM7 appeared clustered and more dispersed and yielded some spots with more intense signals (see **Figure 5**). We then employed LM20, which also recognizes highly methyl-esterified HG, to confirm the labeling pattern observed with JIM7 (Verhertbruggen et al., 2009); the *gosamt1* and *gosamt2* mutants produced lower signals than the wild-type, resulting in different distribution patterns (see **Figure S5**). Similar to JIM7, LM20 detected clustered spots closer to the seed coat with more intense signals, which were also observed closer to the seed coat. Overall, labeling seeds with JIM7 and LM20 confirmed epitopes recognized by these antibodies are less abundant in *gosamt1* and *gosamt2*, which is consistent with the lower content of methyl-esterified HG presented above.

**Figure 6:**
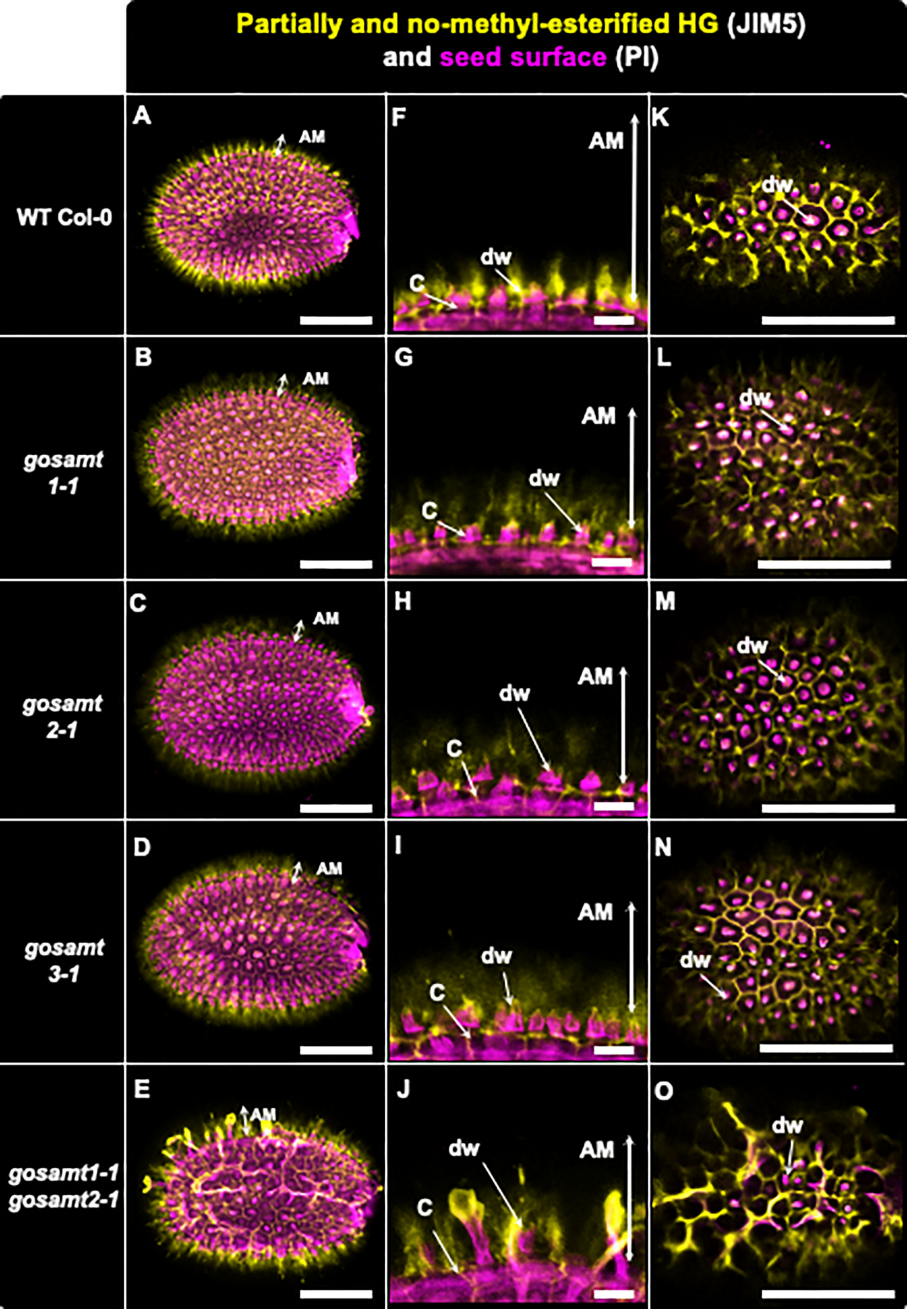
Distribution of low methyl-esterified homogalacturonans recognized by JIM5 antibody in adherent mucilage layers of wild-type and *gosamts* mutants. Confocal microscopy images of AM released from WT Col-0 and *gosamts* mutants mature imbibed seeds. Fluorescence corresponds to JIM5 antibody labelling in yellow, and propidium iodide used to detect seed surfaces in magenta. **(A–D)** Different optical planes showing entire seed. **(F–J)** Close-up of columella. **(K–O)** Top view of seed surface. C = columella; dw distal cell wall; vertical arrows in **(F–J)** indicate labelled AM. Scale bars: **(A–E)** and **(K–O)**=200 µm; **(F–J)**= 50 µm.

Because JIM5 detects low and non-esterified HG, labelling wild-type seeds with this antibody yielded a strong signal that was close to the seed surface and distributed around the columella (Willats et al., 2000). An analysis of the single mutants showed that JIM5 gave a similar signal but was more diffuse than the wild-type (see **Figure 6**). Interestingly, the pattern in the *gosamt1-1gosamt2-1* mutant was different: A strong signal was observed, and signal redistribution was evident and could also be detected at the top of altered structures that resembled columella projections. Notably, LM19, which recognizes low methyl-esterified HG, also emitted a signal near the seed surface and around the columella of the wild-type seeds (see **Figure S6**) (Verhertbruggen et al., 2009). Even though the labelling pattern was similar but with some spots of higher intensity in seeds from the *gosamt* single mutants, a stronger signal located at the top of structures projecting out of the columella was observed in seeds from the *gosamt1-1gosamt2-1* double mutant.

**Figure 7:**
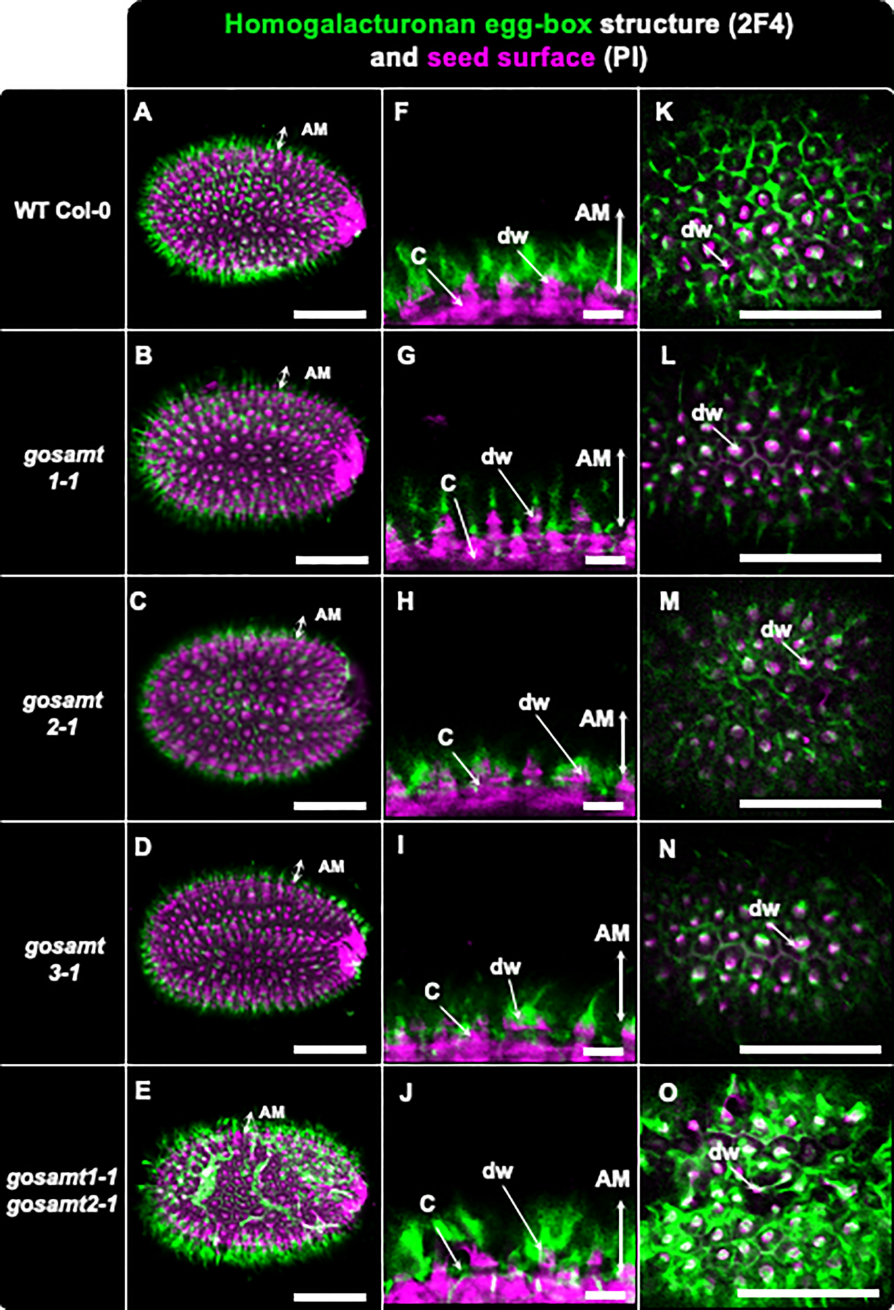
Distribution of egg-box structure recognized by 2F4 antibody in adherent mucilage layer of wild-type and *gosamts* mutants. Confocal microscopy images of AM released from WT Col-0 and *gosamts* mutant mature imbibed seeds. Fluorescence corresponds to the 2F4 antibody labelling in green, and propidium iodide, used to detect the seed Surface, in magenta. **(A–E)** Different optical planes showing entire seed. **(F–J)** Close-up of columella. **(K–O)** Top view of seed surface. C = columella; dw = distal cell wall; vertical arrow highlights labelled AM adherent mucilage. Scale bars: **(A–E)** and **(K–O)** = 200 µm; **(F–J)** = 50 μm.

Non-esterified carboxyl groups allow the dimerization of HG in the presence of calcium. These egg-box structures, which are present in cell walls, can be recognized by 2F4, an antibody that yields a labelling pattern close to the seed surface in the same region where JIM5 detected the low methylated HG in wild-type seeds (see **Figure 7**) (Liners et al., 1989; Temple et al., 2022). Moreover, 2F4 in *gosamt1* showed a different labelling pattern on top of the columella, and the double mutant *gosamt1-1gosamt2-1* exhibited patches of intense signal labelled by this antibody in a less organized pattern than the wild-type; this suggests an increased number of these structures are located close to the *gosamt1-1gosamt2-1* seed coat.

### 3.5 HG methyl-esterification changes not explained by different pectin methylesterase activity

**Figure 8:**
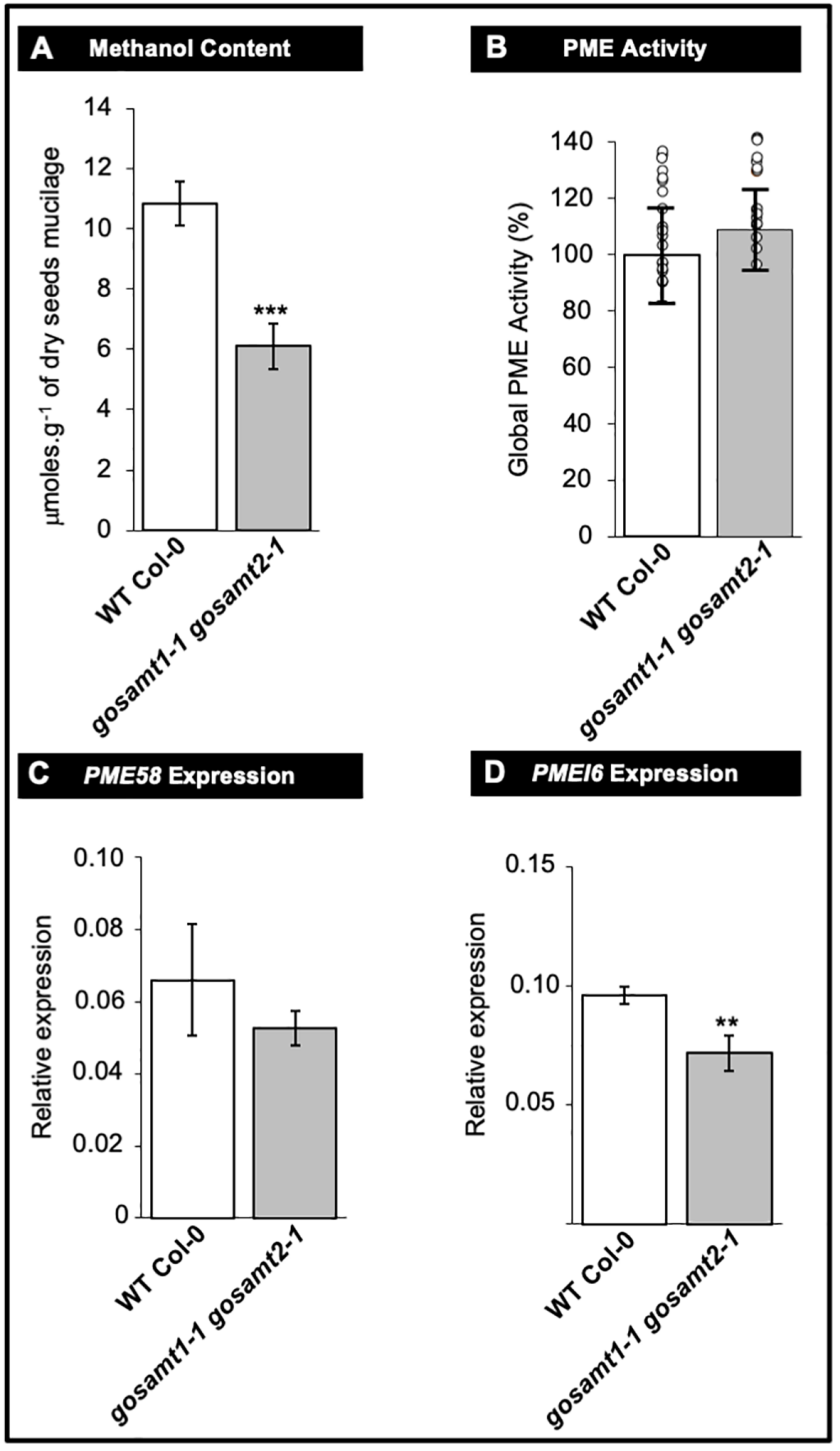
Pectin methyl-esterase (PME) metabolism remains unaffected in *gosamt1-1gosamt2-1* double mutant mature seed. **(A)** Methanol content of soluble plus adherent mucilage layers determined by colorimetric assay after saponification. Mean values are represented with standard error of *n* = 15 from three biological replicates; statistical difference observed during Mann-Whitney analysis with *** p <0.001. **(B)** Global PME activity measured using total protein extract from nature dry seed. Mean PME activity in WT Col-0 normalized to 100%; data presented as mean values ±SD from three biological replicates (*n* = 18); each circle represents WT Col-0 and *gosamt1-1gosamt2-1* double mutant measurement value; no statistical difference observed during *t*-test analysis. **(C, D)** *PME58* and *PME16* expressions measured in mature dry seeds. qRT-PCR analysis used to determine *PME58* and *PMEI6* transcript levels in mature seeds. Steady state mRNA levels are represented as percentage of constitutive *EF1a4* and *seed reference* (At4g12590) genes. Error bars represent SE values from three biological replicate (*n* = 9); Mann-Whitney *U* test with the wild type with **<0.05.

Levesque-Tremblay et al. (2015) concluded PME and PMEI activity regulate the HG methyl-esterification status. In the present study, we observed a significant decrease in the amount of methanol released in the *gosamt1-1gosamt2-1* soluble and adherent mucilage layers (see **Figure 8A**). We documented global PME activity in wild-type and *gosamt1-1gosamt2-1* seeds to determine whether changes leading to lower HG methyl-esterification were the consequence of increased PME activity, but we did not observe significant differences in this activity (see **Figure 8B**); this implies methyl-esterification changes in *gosamt1-1gosamt2-1* seeds are not caused by increased global PME activity.

We also investigated whether the *PME58* and *PMEI6* transcripts, the main players reported so far to play a role in de-methyl-esterification in mucilage, exhibit differences in the double mutant. Even though the q-PCR analyses revealed some decreases in the transcript levels of both genes; the higher variability of the *PME58* analysis yielded no statistical difference between wild-type and *gosamt1-1gosamt2-1* seeds (see **Figures 8C, D**). It is worth noting that these findings do not support that the most significant changes in HG methyl-esterification of this double mutant were the consequence of a greater amount of PME activity.

### 3.6 *GoSAMT* mutation alters adherent mucilage partitioning and cellulose organization

**Figure 9:**
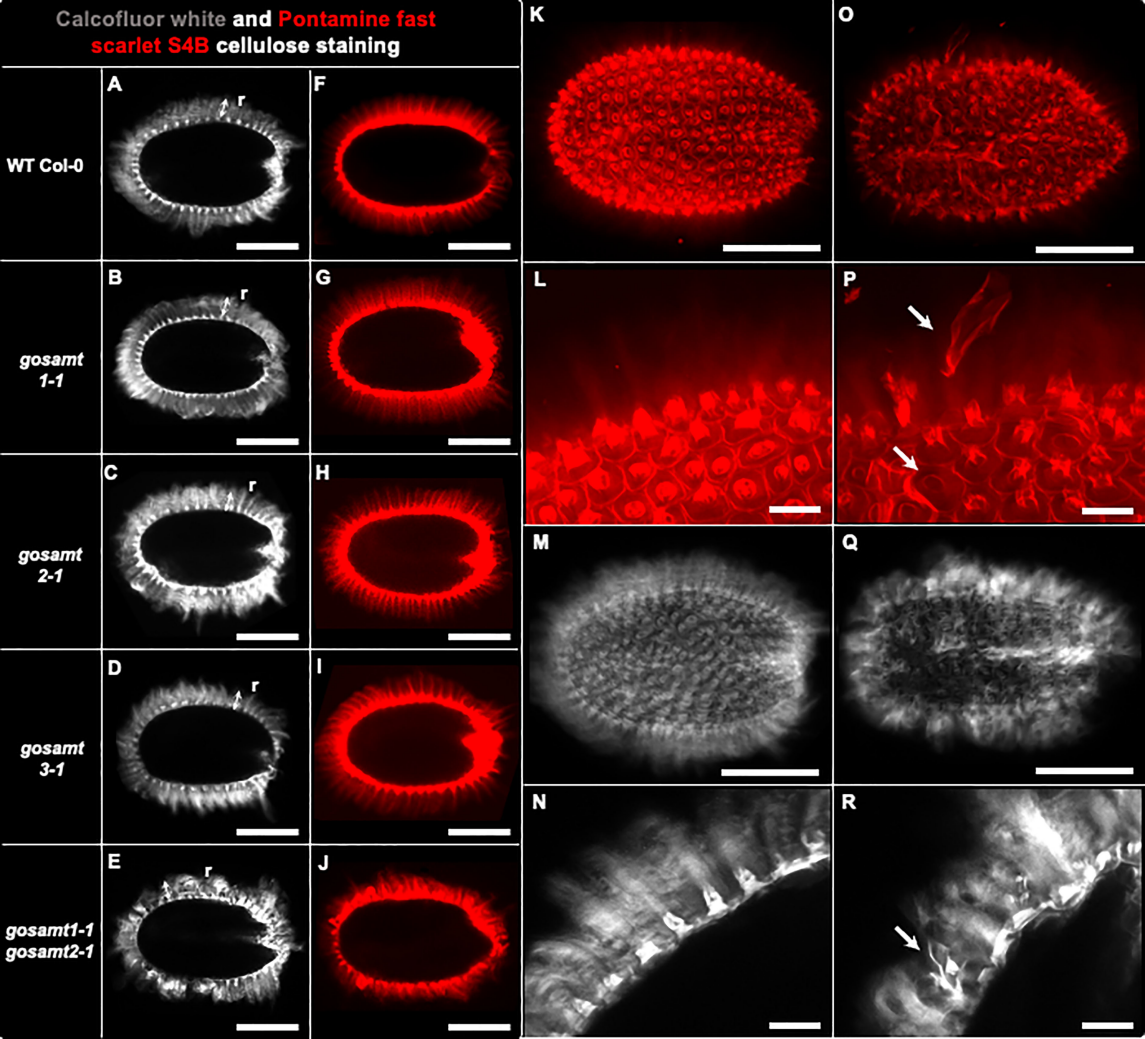
*gosamt1-1gosamt2-1* seed coat epidermal cells exhibit an altered cellulose structure. Confocal microscopy optical sections of B-glycans staining in the inner layer of AM with Calcofluor white (i.e., **A–E, M, N, Q, R**) and Pontamine fast scarlet 4B (S4B) (i.e., **F–L, O, P**). r, two headed arrow, cellulosic rays; white arrow in **(P, R)** show abnormal structure observed in *gosamt1-1gosamt2-1* double mutant. Scale bars: **(A–J)** = 200 µm; **(K, M, O, Q)** = 200 µm; **(L, N, P, R)** = 50 μm.

Since the analyses of the seed surface in the *gosamt1-1gosamt2-1* genotype showed abnormal structures, we investigated the adherent mucilage distribution of cellulose in the mutants using Calcofluor and S4B (Macquet et al., 2007; Harpaz-Saad et al., 2011). While a regular pattern of cellulose stained with Calcofluor and S4B was observed in wild-type seeds, this pattern was distorted in the double mutant, and the columella did not have the shape observed in wild-type seeds (see **Figure 9**).

**Figure 10:**
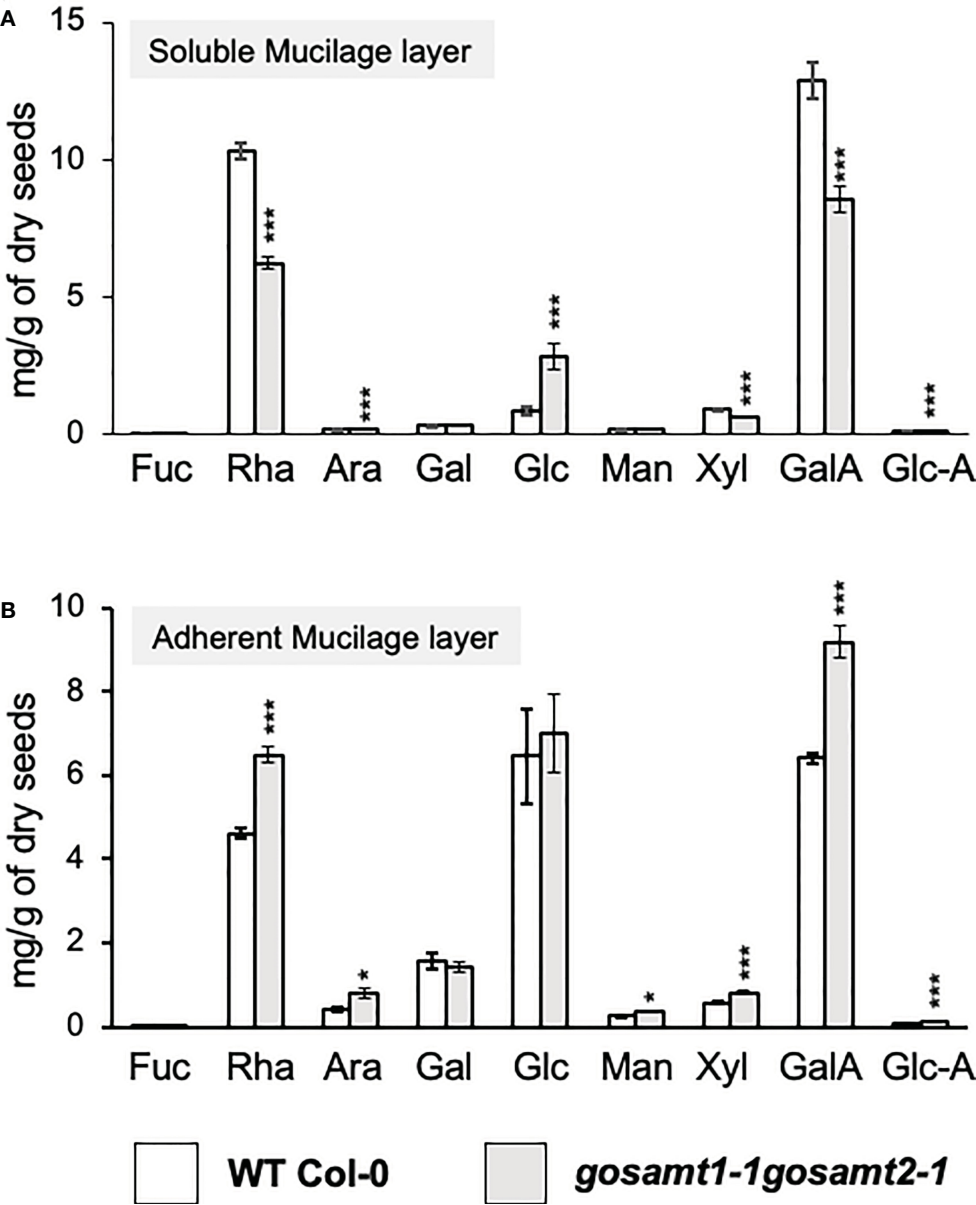
Monosaccharide content in mucilage layers of wild-type and *gosamt1-1 gosamt2-1* mature dry seeds. Quantification of the monosaccharide composition in SM **(A)** and AM **(B)** fractions using HPAEC-PAD. Error bars represent SE from three biological replicates (*n* = 12). Significant differences between genotypes are presented in asterisks. Statistical analyses were performed by using the Mann-Whitney test (*P < 0.05; ***P < 0.001).

Finally, because we observed several changes in the cell surface, we analyzed the monosaccharide composition in the polysaccharide fraction from both mucilage layers of wild-type and *gosamt* mutants (see **Table S3**, **Figure 10**). The *gosamt1-1gosamt2-1* soluble mucilage contained lower levels of GalA and Rha and decreased Xyl content; this confirms lower levels of xylan, a polysaccharide attached to RG-I in mucilage, in the soluble mucilage of this double mutant (Ralet et al., 2016). Analyses of the adherent mucilage showed an increment of the monosaccharides GalA, Rha, and Xyl in *gosamt1-1gosamt2-1*; interestingly, the Ara content was also higher in the AM layer of this mutant. The analysis of the total sugar content in the SM and AM layers did not change, which suggests the different monosaccharide content in each layer is likely due to an altered partitioning of polysaccharides, in particular RG-I, in the *gosamt1-1gosamt2-1* mutant line.

Considering the elevated Ara levels, especially in the adherent mucilage, we conducted a immunodot-blot analysis using the LM30 antibody, which recognizes arabinogalactan-protein, to determine if this was due to increased AGPs in this layer (Wilkinson et al., 2017). This evaluation detected an increment in the signal from this antibody (see **Figure S3**), which confirms higher levels of AGPs in the *gosamt1-1gosamt2-1* AM layer.

## 4 Discussion

*GoSAMT*s are transporters involved in methylation reactions occurring in the Golgi apparatus. In the epidermal cells of seed coats, most biosynthetic activity in the Golgi is dedicated to biosynthesizing polysaccharides comprising the mucilage (Young et al., 2008). RG-I is the most abundant polymer, HG accounts for approximately 10% of the polysaccharides, and low amounts of hemicelluloses are also present (Voiniciuc et al., 2015). Because mucilage methyl groups are primarily located in HG, impaired delivery of SAM into the Golgi would lessen HG methyl-esterification during biosynthesis of this polymer. After evaluating methanol release, testing methyl-esterified epitopes *via* dot blot, and performing immunofluorescence analyses, our findings support this hypothesis and confirm the importance of *GoSAMT*s in the biosynthesis of methyl-esterified HG.

S-Adenosylmethionine is utilized by HG–methyl-transferases to produce highly methyl-esterified HG (Ibar and Orellana, 2007). Du et al. (2020) showed that *QUA2* is involved in the methyl-esterification of HG; however, it is likely that other HG methyl-transferases might be involved. In this regard, co-expression analyses showed that several putative methyl-transferases co-express with *GoSAMT1* and *GoSAMT2* (Temple et al., 2022). Interestingly, we found eight of these genes expressed in seed coats at a stage of development that matched the biosynthesis of mucilage (see **Figure S7**); this implies several methyl-transferases may act during HG biosynthesis, and each could play different roles during HG methyl-esterification. By the end of the biosynthetic process, the HG becomes highly methyl-esterified (Mouille et al. (2007); however, it remains unknown whether methylation is random or if methyl-esterification patterns are created with the addition of multiple methyl groups.

Upon secretion, HG is subjected to demethyl-esterification by the coordinated action of PMEs and their inhibitors, PMEIs, and a 30–34% degree of methylation is achieved in the mucilage (Saez-Aguayo et al., 2013; Voiniciuc et al., 2015). In the present study, we concluded the lower HG methyl-esterification observed in *gosamt1-1gosamt2-1* was not caused by increased PME activity; as such, the mutants in these transporters offer an unprecedented tool to study how altering the most upstream Golgi component involved in HG methyl-esterification affects this process.

We detected similar HG levels in both the *gosamt1-1gosamt2-1* and the wild-type, in spite of the lower methyl-esterification that exhibit the polymer in this mutant; this interesting finding should be further investigated in future research. While we do not yet know if the size and number of HG molecules are similar in the cell walls of the wild-type and mutant phenotypes, our findings pose a question regarding the coordination between the biosynthesis of HG with its methyl-esterification. HG is highly methyl-esterified in the Golgi, and while some have hypothesized the stability of this modification is important until it reaches the cell wall, our results lead to questions about whether HG can be synthesized and exported to a cell wall regardless of the degree of methyl-esterification in the Golgi apparatus. In this regard, experiments conducted on the wild-type and mutant alleles to differentiate between methyl-esterified and demethyl-esterified HG in the Golgi with the use of immunogold and an electron microscopy would help to address this question.

There is substantial evidence that methyl-esterified HG domains play functional roles in mucilage (Francoz et al., 2019; Dauphin et al., 2022). An interesting example is the finding that PMEI6 creates a partially demethyl-esterified pattern and allows the positioning of peroxidase36, which participates in cell-wall loosening in mucilage during early development (Francoz et al., 2019). Using JIM7 and LM20 to recognize highly methyl-esterified HG provided a regular labeling pattern resembling a halo located toward the periphery of the AM layer in wild-type seeds (see **Figure S8**); this is in line the findings of Macquet et al. (2007) and Saez-Aguayo et al. (2013) and confirm high HG methyl-esterification domains are more abundant in the outer region of the AM. Notably, however, *gosamt1* and *gosamt2* both yielded a lower signal when these antibodies were utilized, which confirms HG in these mutants is less methylated. Even though the *gosamt1-1gosamt2-1* adherent mucilage exhibited the most significant decrease in methanol release, it did not produce a weaker signal than the single mutants; instead, clusters and spots with high-intensity signals were observed but were more widely dispersed (see **Figure S8**); this indicates epitopes recognized by the antibodies were rearranged and suggests the methyl-esterified domains remaining in the double mutant become clustered after the cell-wall breakage. Furthermore, the signal provided by JIM7 and LM20 was closer to the seed surface, which suggests highly methyl-esterified HG domains were closer to the seed coat in the double mutant than in the wild-type (see **Figure S8**).

The antibodies JIM5 and LM19 recognize low HG methyl-esterified epitopes. These antibodies detected HG epitopes in regions around the columella and close to the seed coat in the wild-type seeds; in contrast, this region yielded virtually no signal of highly methyl-esterified HG. The signal provided by JIM5 and LM19 was higher in the *gosamt1-1gosamt2-1*, which is consistent with the lower methyl-esterification of the HG in the *GoSAMT* double mutant. Interestingly, after employing JIM5 to analyze different pectin methyl-transferase mutant alleles, Du et al. (2022b) similarly concluded the antibody produced a stronger signal in the *tfa2* and *tsd2* mutant-allele mucilage. Furthermore, the distorted JIM5 and LM19 labeling patterns in the *gosamt1-1gosamt2-1* double mutant infer HG epitopes in the AM layer were reorganized (see **Figure S8**).

A greater number of regions with lower HG methyl-esterification promote coordination with calcium ions and form egg-box structures close to the seed coat. This suggests the region near the seed coat exhibits a stiffer matrix. 2F4 recognized the egg-box structures, and clear labeling was observed around the columella in the wild-type seeds. The analysis of the *gosamt1-1gosamt2-1* mutant using 2F4 showed a stronger signal, which supports the hypothesis that an increase in low methyl-esterified HG should lead to an increase in egg-box structures; moreover, this finding also confirms the double mutant had a stiffer cell wall near the seed coat than that of the wild-type.

Mutants exhibiting lower levels of methyl-esterification demonstrate delayed mucilage extrusion (Rautengarten et al., 2008; Saez-Aguayo et al., 2013; Voiniciuc et al., 2013; Du et al., 2022b). We observed the *gosamt1* mutants and the *gosamt1-1gosamt2-1* double mutant all exhibited delayed onset of the mucilage extrusion. Since there were more egg-box structures near the surface of the seed coat in the double mutants, it is likely that the primary walls containing mucilage are more resistant to bursting after seed imbibition. The greater number of long distal walls suggest the walls burst close to the columella, rather than where they normally broke.

Our findings related to monosaccharide composition suggest polysaccharides change their distribution between the SM and AM layers. When Turbant et al. (2016) analyzed the monosaccharide composition of soluble and adherent mucilage extracted in the presence of EDTA in *pme58* mutants, they observed altered polysaccharide distribution, mostly RG-I, and they documented higher levels of GalA and Rha in soluble mucilage and lower content of these monosaccharides in adherent mucilage; this indicated changed polysaccharide partitioning between soluble and adherent mucilage in the *pme58* mutant. In the present study, we detected a different monosaccharide distribution in the soluble and adherent mucilage, and observed *gosamt1-1gosamt2-1* also exhibited different partitioning. In contrast to the results reported by Turbant et al. (2016), however, we observed lower GalA, Rha, and Xyl levels in the soluble mucilage and higher levels of these monosaccharides in the adherent mucilage. After investigating *bxl1-1*, a mutant in a bifunctional xylosidase/arabinofuranosidase that trims arabinan from RG-I, Arsovski et al. (2009) documented lower Rha and Xyl content in the soluble mucilage and higher Ara content in mucilage. Interestingly, the *bxl1-1* mutant exhibits a patchy and delayed mucilage release phenotype, similar to what we observed in *gosamt1-1gosamt2-1*. Furthermore, Williams et al. (2020) reported that in contrast to the wild-type, RG-I extracted from the *bxl1-3* mutant has side chains that could change the molecular interactions of RG-I.

We observed higher levels of Ara in the *gosamt1-1gosamt2-1* adherent mucilage; this correlates with lower levels of Rha, GalA, and Xyl in the soluble mucilage and increased levels of these monosaccharides in the adherent mucilage of this double mutant, which suggests a greater amount of RG-I attaches to the *gosamt1-1gosamt2-1* adherent mucilage. Interestingly, Tan et al. (2023) reported RG-I is covalently linked to AGP in the cell walls of *Arabidopsis*-cultured cells. We observed more AGP is present in the adherent mucilage of the double mutant than in the wild type, which is in line with the hypothesis that higher Ara content is due to increased levels of AG, which may retain RG-I in the adherent mucilage. In this regard, it has been reported that the SOS5 AGP is required for mucilage adherence, and mutants in this gene yield higher RG-I content in soluble mucilage (Griffiths et al., 2016); this reinforces the understanding that AGPs play a role in retaining RG-I in adherent mucilage.

Why does a mutant in the transport of SAM exhibit increased AGP levels in mucilage? Others have demonstrated that mutants in *urgt2*, a UDP–rhamnose transporter needed for RG-I biosynthesis, exhibit significant changes in the seed transcriptome and is likely to compensate for modifications in the cell wall (Parra-Rojas et al., 2019); it is possible a similar response occurs in the *gosamt1-1gosamt2-1* mutant. The results of the present study verify *gosamt* mutants exhibited variations in the polysaccharide molecular interaction, which may arise from changes in such components as Ara and AGP, but they also may occur due to changes in methyl-esterification domains present in HG that cause different breakage of the walls containing mucilage in the seeds from the *GoSAMT* mutants.

## Data availability statement

The raw data supporting the conclusions of this article will be made available by the authors, without undue reservation.

## Author contributions

JP-R, PS-O, SS-A, HT, PD, and AO conceived and designed the study. JP-R and DS conducted the biochemical experiments, and HS-G assisted. PS-O performed all microscopical analyses. JP-R, PS-O, HS-G, SS-A, HT, PD, and AO. conducted the data analysis and interpretation. AO wrote the paper with contributions from all authors. All authors contributed to the article and approved the submitted version.
