# Supplementary material for: GoSAMTs are required for pectin methyl-esterification and mucilage release in seed coat epidermal cells
Source: Front Plant Sci. 2023 Feb 8;14:1099573. doi: 10.3389/fpls.2023.1099573 (PMC9946043; doi:10.3389/fpls.2023.1099573)
Supplement: Supplementary file 1 [file DataSheet_1.pdf]

Supplementary Material

GoSAMTs required for pectin methyl-esterification and mucilage release in seed epidermal cells

Juan Pablo Parra-Rojas, Pablo Sepúlveda-Orellana, Dayan Sanhueza, Hernán Salinas-Grenet, Henry Temple, Paul Dupree, Susana Saez-Aguayo, Ariel Orellana[*]

[*]**Correspondence:** Corresponding Author: aorellana@unab.cl

1 Supplementary Figures and Tables

1.1 Supplementary Figures

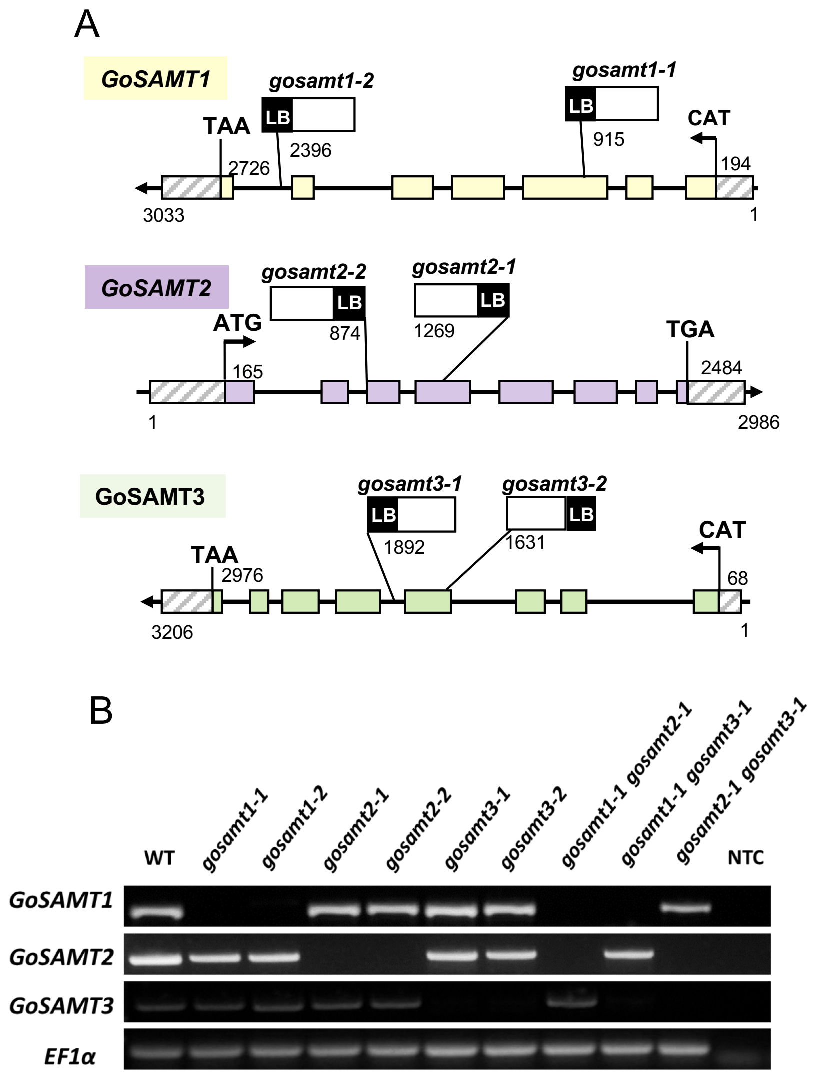

Supplementary Figure 1. Genetic characterization of *gosamt* mutant lines. (A) Schematic representation of *GoSAMTs* gene structure as annotated by the Arabidopsis Information Resource (http://www.arabidopsis.org). The sites and orientation of insertion lines in *gosamt1*, *gosamt2*, *gosamt3* lines are indicated by the presence of left border (LB). Numbers indicate the position of different features in bp. Colored blocks represent exon, hatched bars represent 5'and 3' UTR and white black lines represent intron sites. (B) Effect of the *gosamt* mutation on *GOSAMTs* expression. RT-PCR analysis was performed with specific primers amplifying the whole length *GOSAMTS* CDS. A control amplification was performed with primers for EF1a4. All PCR reactions were carried out using 30 cycles. All primers utilized in the present study are listed in Supplementary Table 1.

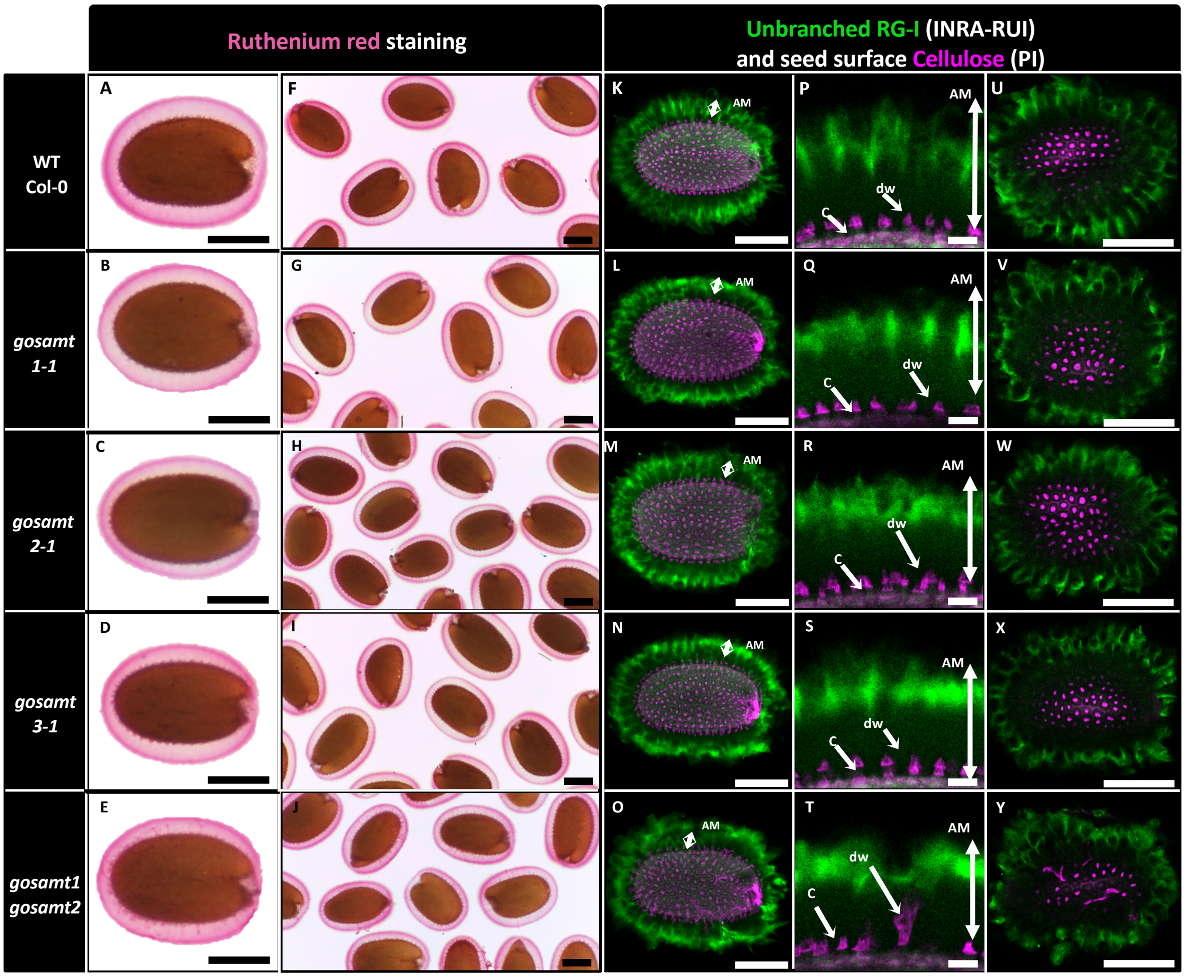

Supplementary Figure 2. Adherent mucilage structure and RG-I labelling with INRA-RU1. (A–J) Ruthenium red staining of WT Col-0; *gosamt1-1*, *gosamt2-1*, *gosamt3-1*; and *gosamt*1-1*gosamt*2-1 double-mutant seeds imbibed in water. (K–Y) Confocal microscopy optical sections of unbranched RG-I epitopes in AM released from WT Col-0 and *gosamt* mutants using the monoclonal antibody INRA-RU1 (green), and propidium iodide for seed surface staining (magenta) . C = columella; dw = distal cell wall; AM with vertical arrow = labelled adherent mucilage. Scale bars: A–E, K–O, and U–Y = 200 µm; F–J and P–T = 50 µm.

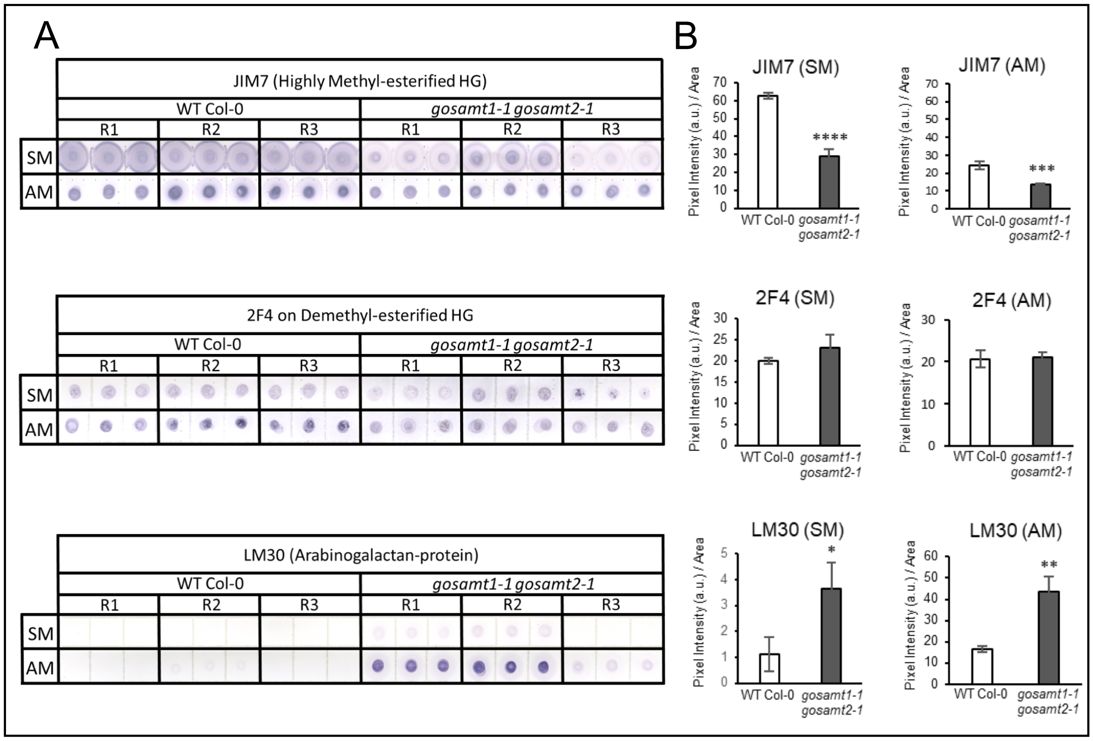

Supplementary Figure 3. Presence of pectin and AGP epitopes on WT-Col-0 and *gosamt1-1 gosamt2-1 s*oluble and adherent mucilage. (A) Immunodot blots were performed from the same SM and AM samples used in the monosaccharide analysis. (B) Dot blot pixel intensity quantified. Error bars represent SE from three technical replicates of three biological repeats (n=9). Asterisks indicate significant statistical differences using *t*-test where *a* = 0.05 (**P* < 0.05, ***P* < 0.005, ****P* < 0.0005, *****P* < 0.00005).

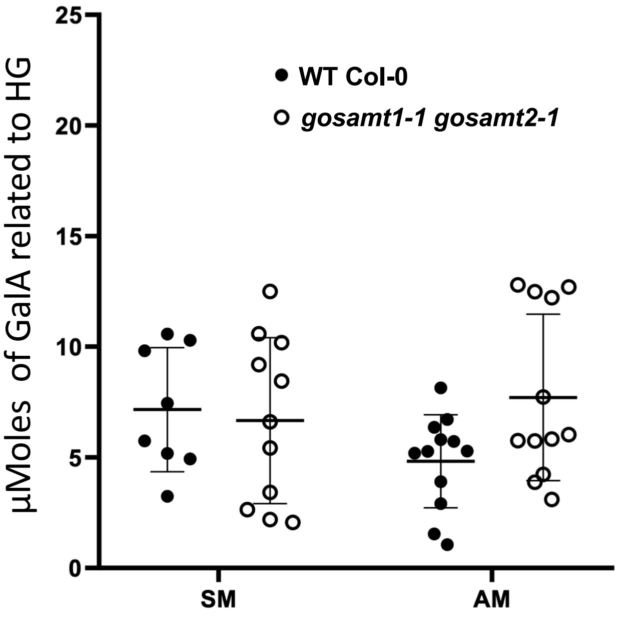

Supplementary Figure 4. Galacturonic acid related to HG is not affected in the *gosamt1-1 gosamt2-1* double mutant. Molar subtraction between galacturonic acid (GalA) and rhamnose was employed to estimate the HG level in mucilage from WT Col-0 and *gosamt1-1gosamt2-1* double mutant. SM and AM represent soluble mucilage and adherent mucilage fractions, respectively. Data are presented as mean values ± S.D. from three biological replicates. Black circles and white circles represent values of each measurement in WT-Col-0 and *gosamt1-1gosamt2-1* double mutant, respectively. No statistical difference was observed using the Mann-Whitney test.

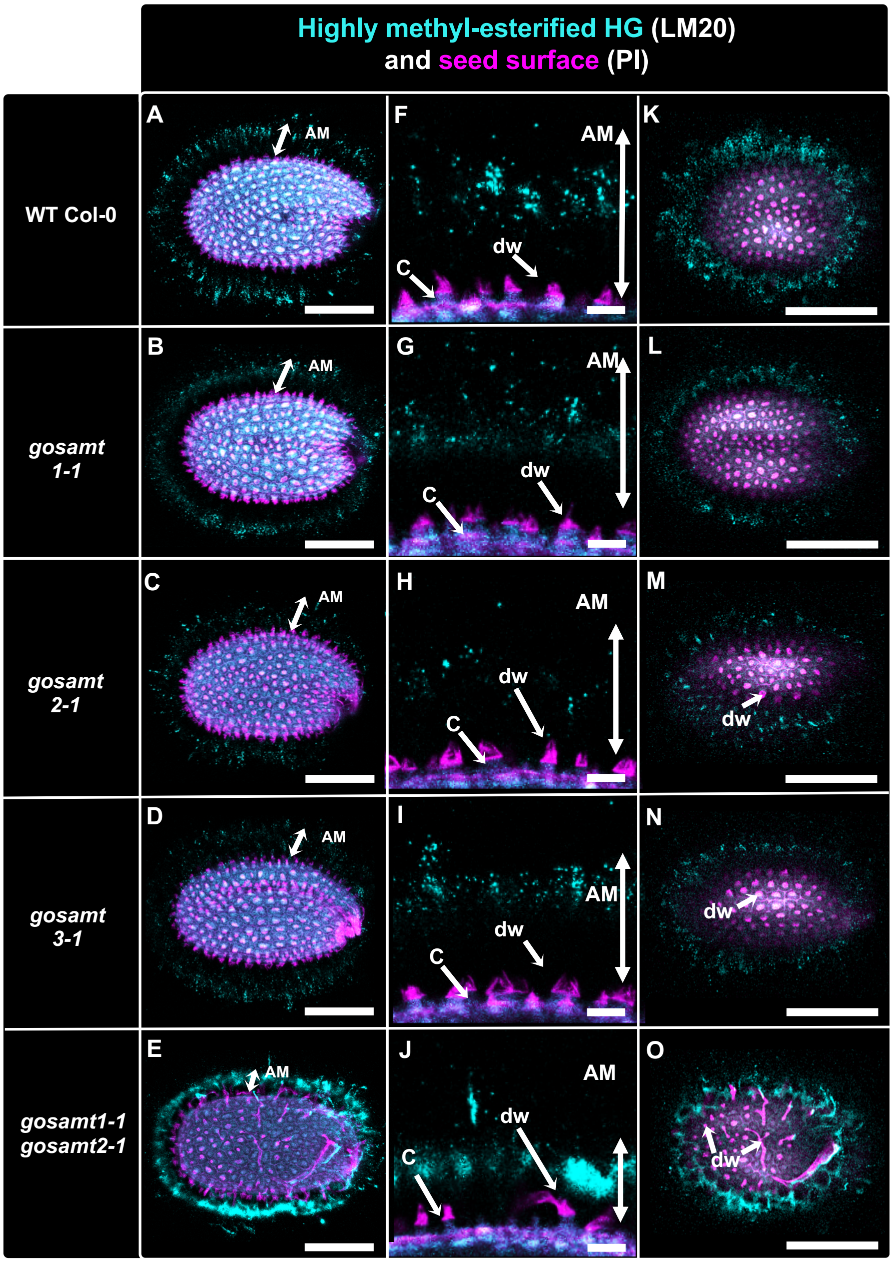

Supplementary Figure 5. Distribution of highly methyl-esterified homogalacturonan recognized by LM20 antibody in adherent mucilage layer of wild-type and *gosamts* mutants mature dry seeds. Confocal microscopy optical sections of AM released from WT Col-0 and *gosamt* mutants mature imbibed seeds. Fluorescence corresponding to LM20 antibody labelling in cyan, and propidium iodide, used to detect the seed surface in magenta. (A–E) Different optical planes showing the whole seed. (F–J) Close-up of columella. (K–0) Top view of seed surface. C = columella; dw = distal cell wall; AM with vertical arrow = labelled adherent mucilage. Scale bars: A–E and K–O = 200 µm; F–J = 50 µm.

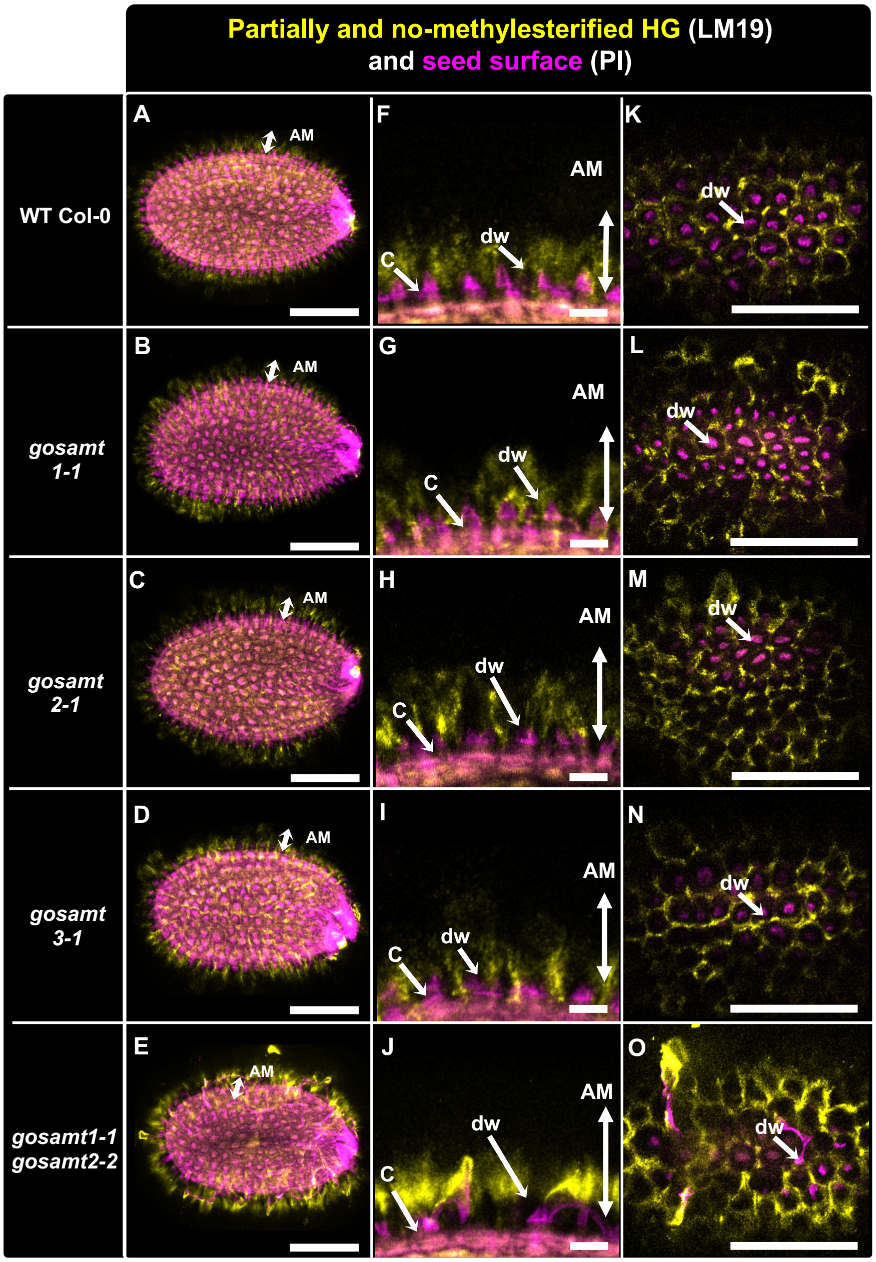

Supplementary Figure 6. Distribution of low methyl-esterified homogalacturonan recognized by LM19 antibody in adherent mucilage layer of wild-type and *gosamt* mutants mature dry seeds. Confocal microscopy optical sections of AM released from WT Col-0 and *gosamt* mutants mature imbibed dry seeds. Fluorescence corresponds to the LM19 antibody labelling in yellow, and propidium iodide, used to detect the seed surface, in magenta. (A–E) Different optical planes showing the whole seed. (F–J) Close-up of columella. (K–O) Top view of seed surface. C = columella; dw = distal cell wall; AM with vertical arrow = labelled adherent mucilage. Scale bars: A–E and K–O = 200 µm; F–J = 50 µm.

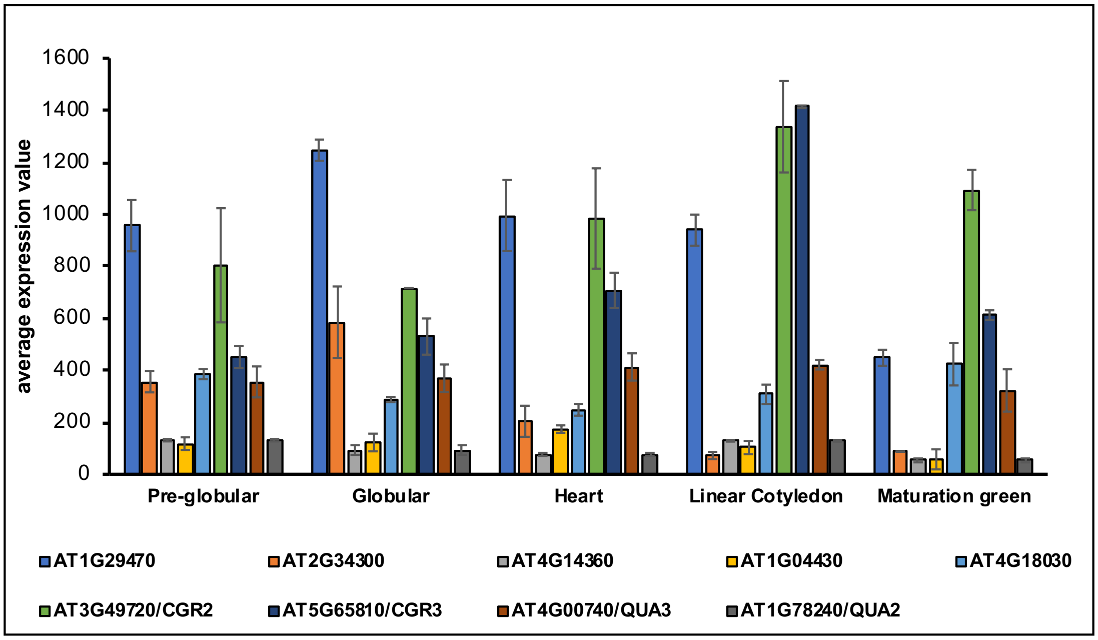

Supplementary Figure 7. Expression levels of putative S-Adenosylmethionine-dependent methyl transferases in seed coat integument. Putative Pectin Methyl Transferases co-expressing with *GoSAMT1* and *GoSAMT2*, according to Temple et al. (2022), were analysed and those expressed in the seed coat, identified. Seed coat expression values during seed development were obtained from the eFP browser.

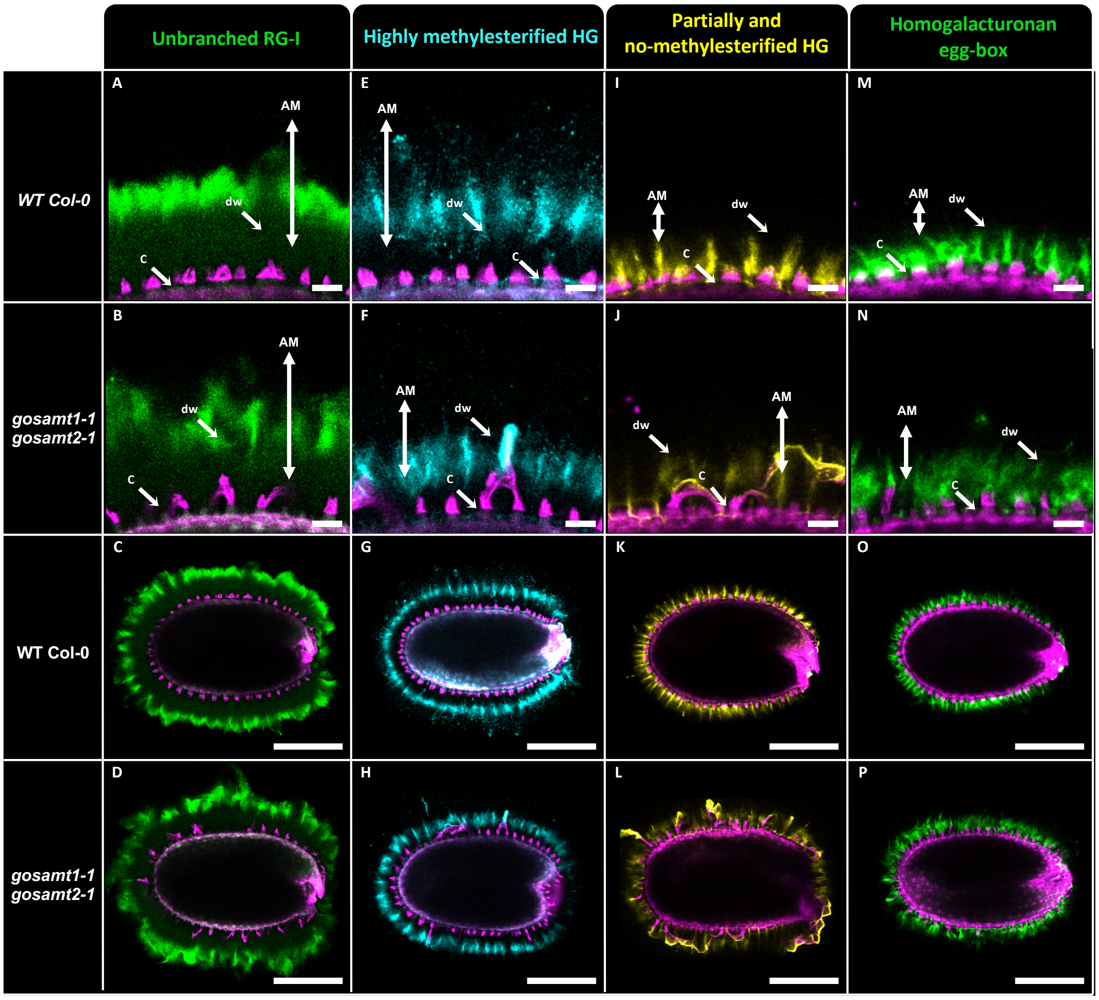

Supplementary Figure 8. Summary of global changes in HG epitopes distribution between wild-type and *gosamt1-1 gosamt2-1* double mutant. Confocal microscopy of optical sections of AM released from WT Col-0 and *gosamt1-1gosamt2-1* double mutant from dry seeds. Fluorescence corresponding to INRA-RU1 antibody (green), highly methylesterified HG epitope of JIM7 antibody (cyan), partially and no-methylesterified HG epitope of JIM5 antibody (yellow) and HG egg-box structure epitope of 2F4 antibody (green). Propidium iodide, used to detect seed surface in magenta. C = columella; arrows indicate different epitope pattern distributions. Scale bars: A, B, E, F, I, J, M, and N = 50 µm; C, D, G, H, K, L, O, and P = 200 µm.

1.2 Supplementary Tables

Supplemental Table 1. Primers used in present study.

**Expression Analysis (5' to 3') Primers**	
GoSAMT1 Forward	TTCGAAGGCAGAGAAAAGGA
GoSAMT1 Reverse	TTGCTGGTTTTGGATCTTCC
GoSAMT2 Forward	GTGCTGCACTATTGCTTCCA
GoSAMT2 Reverse	GGATGGCCAAAACAGTCCTA
GoSAMT3 Forward	GTTTGGGCTAAGAGCTGGTG
GoSAMT3 Reverse	TGGAGATCCATCTGACAGCA
EF1α Forward	TCACCCTTGGTGTCAAGCAGAT
EF1α Reverse	CAGGGTTGTATCCGACCTTCTT
At4g12590 Forward	TGGCATTGACTTGAGCACTGTCG
At4g12590 Reverse	TCGAGGTAGTGCCCATTCGTGCT
PME58 Forward	GGGGTTGATGGTGAACTGAA
PME58 Reverse	TTGTGATGCCAACAGCGACAAC
PMEI6 Forward	GGCAGATAAGCGATCTCGCCAC
PMEI6 Reverse	AGCCAGAGCATTGCTGCATAGTC
**RT-PCR (5' to 3') Primers**	
EF1α Forward	ATGCCCCAGGACATCGTGATTTCAT
EF1α Reverse	TTGGCGGCACCCTTAGCTGGATCA
GoSAMT1 Forward	TGGGTTTAGAGGGTCAGCTT
GoSAMT1 Reverse	ATGGAGATCTTCTACTTCGT
GoSAMT2 Forward	ATGGAGATTTTCTACTACTT
GoSAMT2 Reverse	TATGTTGAGGGGATCTTCTT
GoSAMT3 Forward	GAGGGTAAGAGGATCAACTT
GoSAMT3 Reverse	ATGGAGGTTTTCTACTACTT
**Genotyping (5' to 3') Primers**	
GoSAMT1 Forward	AACGAGAGGCAATGGAGCTA
GoSAMT1 Reverse	GTGTTTCGGCTGCATCATTA
GoSAMT2 Forward	CACGCTGCTTCAGATTCGTA
GoSAMT2 Reverse	GAAACCATGGGGAATCTCCT
GoSAMT3 Forward	GAAGGTTGCAAAGACGAAGC
GoSAMT3 Reverse	TCGGTTTCTTTTGGTTTGGA
o8409	ATATTGACCATCATACTCATTGC
LBb1.3	ATTTTGCCGATTTCGGAAC

Supplemental Table 2. Percentage of seed mucilage extrusion in wild-type and *gosamts* mutant seeds imbibed in Ruthenium red staining.

Genotype	Non-extrusion (%)	Extrusion (%)
WT (Col-0)	4.5	95.5
GoSAMT1-1	6.9	93.1
GoSAMT1-2	21.9	78.1
GoSAMT2-1	8.2	91.8
GoSAMT2-2	9.5	90.5
GoSAMT3-1	5.4	94.6
GoSAMT3-2	6.7	93.3
GoSAMT1-1–GoSAMT2-1	35.7	64.3
GoSAMT1-1–GoSAMT3-1	5.4	94.6
GoSAMT2-1–GoSAMT3-1	10.0	90.0

Supplemental Table 3. Monosaccharide composition of adherent and soluble mucilage layers in wild-type and *GoSAMT* mutant seeds.

`	WT Col-0	*GoSAMT1-1*	*GoSAMT1-2*	*GoSAMT2-1*	*GoSAMT2-2*	*GoSAMT3-1*	*GoSAMT3-2*	*GoSAMT1-1–GoSAMT2-1*	*GoSAMT1-1–GoSAMT3-1*	*GoSAMT2-1–GoSAMT3-1*
Soluble Mucilage										mg/g of dry seeds
Gal-A	12.87 (0.642)	11.42 (0.496)	12.69 (0.734)	14.22 (1.428)	13.51 (0.596)	12.44 (0.406)	13.35 (0.381)	8.55 (0.454)***	13.50 (0.436)	11.25 (0.467)
Rha	10.30 (0.273)	9.28 (0.198)*	10.59 (0.419)	11.01 (0.817)	10.73 (0.363)	9.38 (0.195)*	10.56 (0.252)	6.22 (0.236)***	10.67 (0.299)	8.04 (0.257)***
Fuc	0.02 (0.001)	0.02 (0.001)	0.02 (0.001)	0.02 (0.001)	0.02 (0.001)	0.02 (0.001)	0.02 (0.001)	0.02 (0.001)	0.02 (0.001)	0.027 (0.002)
Ara	0.15 (0.005)	0.14 (0.003)	0.15 (0.007)	0.18 (0.008)*	0.15 (0.005)	0.16 (0.017)	0.14 (0.003)	0.20 (0.008)***	0.15 (0.006)	0.16 (0.014)
Xyl	0.95 (0.005)	0.93 (0.030)	0.94 (0.030)	1.11 (0.097)	0.95 (0.017)	0.95 (0.028)	0.94 (0.022)	0.64 (0.030)***	0.97 (0.024)	0.85 (0.035)
Man	0.17 (0.004)	0.15 (0.006)*	0.18 (0.012)	0.19 (0.013)	0.18 (0.008)	0.15 (0.004)**	0.18 (0.008)	0.18 (0.013)	0.18 (0.009)	0.14 (0.007)*
Gal	0.33 (0.007)	0.32 (0.012)	0.34 (0.013)	0.39 (0.026)	0.35 (0.006)	0.34 (0.013)	0.34 (0.010)	0.35 (0.019)	0.36 (0.011)	0.33 (0.020)
Glc	0.85 (0.124)	0.73 (0.061)	0.63 (0.039)	0.93 (0.099)	0.71 (0.061)	0.51 (0.052)	0.66 (0.076)	2.82 (0.441)***	0.58 (0.037)	0.75 (0.108)
Glc-A	0.09 (0.010)	0.14 (0.015)*	0.09 (0.009)	0.17 (0.014)***	0.10 (0.015)	0.16 (0.010)***	0.09 (0.007)	0.14 (0.010)***	0.10 (0.009)	0.13 (0.007)**
**Total sugars (SM)**	**25.77 (1.076)**	**23.17 (0.826)**	**25.69 (1.261)**	**28.26 (2.505)**	**26.77 (1.074)**	**24.14 (0.729)**	**26.33 (0.763)**	**19.17 (1.217)*****	**26.58 (0.836)**	**21.72 (0.921)****

Sugars	WT Col-0	*GoSAMT1-1*	*GoSAMT1-2*	*GoSAMT2-1*	*GoSAMT2-2*	*GoSAMT3-1*	*GoSAMT3-2*	*GoSAMT1-1–GoSAMT2-1*	*GoSAMT1-1–GoSAMT3-1*	*GoSAMT2-1–GoSAMT3-1*
Adherent Mucilage										mg/g of dry seeds
Gal-A	6.42 (0.118)	7.34 (0.195)***	6.58 (0.280)	7.94 (0.277)***	6.72 (0.173)	6.65 (0.168)	7.24 (0.319)	9.18 (0.383)***	7.45(0.292)**	7.32 (0.245)**
Rha	4.62 (0.108)	4.96 (0.148)	5.28 (0.104)***	5.87 (0.260)***	4.61 (0.124)	4.72 (0.184)	4.94 (0.142)	6.49 (0.183)***	5.48(0.164)***	5.13 (0.125)**
Fuc	0.04 (0.003)	0.04 (0.002)	0.04 (0.001)	0.04 (0.001)	0.04 (0.002)	0.04 (0.002)	0.04 (0.001)	0.04 (0.002)	0.04(0.004)	0.04 (0.002)
Ara	0.42 (0.049)	0.45 (0.061)	0.36 (0.028)	0.46 (0.038)	0.37 (0.024)	0.31 (0.029)	0.39 (0.035)	0.79 (0.114)*	0.35(0.018)	0.42 (0.031)
Xyl	0.57 (0.029)	0.66 (0.023)*	0.60 (0.025)	0.68 (0.022)**	0.61 (0.015)	0.60 (0.017)	0.62 (0.020)	0.82 (0.041)***	0.64(0.018)	0.67 (0.020)**
Man	0.26 (0.030)	0.33 (0.028)*	0.29 (0.035)	0.32 (0.024)	0.28 (0.019)	0.29 (0.034)	0.29 (0.024)	0.37 (0.029)*	0.34(0.026)*	0.30 (0.016)
Gal	1.56 (0.196)	1.43 (0.137)	1.33 (0.127)	1.52 (0.085)	1.31 (0.087)	1.22 (0.106)	1.38 (0.109)	1.43 (0.126)	1.61(0.152)	1.36 (0.027)
Glc	6.45 (1.140)	5.41 (1.052)	5.35 (1.031)	5.57 (0.726)	4.51 (0.550)	4.22 (0.849)	4.94 (0.142)	7.00 (0.931)	7.35(1.366)	4.92 (0.130)
Glc-A	0.07 (0.006)	0.09 (0.005)*	0.08 (0.009)	0.085 (0.006)	0.08 (0.004)	0.07 (0.005)	0.08 (0.007)	0.123 (0.007)***	0.08(0.009)	0.08 (0.004)
**Total sugars (AM)**	**20.41 (1.682)**	**20.74 (1.654)**	**19.94 (1.642)**	**22.53 (1.442)**	**18.55 (1.000)**	**18.16 (1.395)**	**19.95 (1.521)**	**26.26 (1.819)***	**23.39(2.052)**	**20.27 (0.603)**
**Total sugars (SM+AM)**	**46.18 (0.864)**	**43.91 (1.226)**	**45.63 (1.129)**	**49.81 (2.505)**	**45.48 (1.088)**	**42.30 (1.144)***	**47.00 (1.54)**	**45.43 (2.27)**	**50.41(0.821)***	**41.99 (1.04)****
**Total sugars (SM+AM) %**	**100 (1.87)**	**95.09 (2.65)**	**98.80 (2.44)**	**107.87 (5.42)**	**98.49 (2.35)**	**91.62 (2.478)**	**101.78 (3.33)**	**98.38 (4.91)**	**109.16(1.77)**	**90.93 (2.25)**
